# Globally discordant Isocrinida (Crinoidea) migration confirms asynchronous Marine Mesozoic Revolution

**DOI:** 10.1038/s42003-018-0048-0

**Published:** 2018-05-17

**Authors:** Rowan J. Whittle, Aaron W. Hunter, David J. Cantrill, Kenneth J. McNamara

**Affiliations:** 1 British Antarctic Survey, High Cross, Madingley Road, Cambridge, CB3 0ET UK; 2 Department of Earth Sciences, University of Cambridge, Downing Street, Cambridge, CB2 3EQ UK; 3 School of Earth Sciences, The University of Western Australia, 35 Stirling Highway, Crawley, WA 6009 Australia; 4 Royal Botanic Gardens Victoria, Birdwood Avenue, Melbourne, VIC 3004 Australia

## Abstract

The Marine Mesozoic Revolution (MMR, starting ~200 million years ago) changed the ecological structure of sea floor communities due to increased predation pressure. It was thought to have caused the migration of less mobile invertebrates, such as stalked isocrinid crinoids, into deeper marine environments by the end of the Mesozoic. Recent studies questioned this hypothesis, suggesting the MMR was globally asynchronous. Alternatively, Cenozoic occurrences from Antarctica and South America were described as retrograde reversions to Palaeozoic type communities in cool water. Our results provide conclusive evidence that isocrinid migration from shallow to deep water did not occur at the same time all over the world. The description of a substantial new fauna from Antarctica and Australia, from often-overlooked isolated columnals and articulated crinoids, in addition to the first compilation to our knowledge of Cenozoic Southern Hemisphere isocrinid data, demonstrates a continuous record of shallow marine isocrinids from the Cretaceous-Paleogene to the Eocene/Oligocene boundary.

## Abstract (web summary)

Rowan Whittle et al. present fossil evidence of new isocrinid (sea lily) species from Antarctica and Australia. They show that isocrinid migration from shallow to deep water occurred at different times across the globe, spanning the Cretaceous-Paleogene to the Eocene/Oligocene.

## Introduction

Interactions between predators and prey have shaped the evolution of life and predation is thought to have been responsible for many major trends in the fossil record^1–3^. During the Marine Mesozoic Revolution (MMR, starting ~200 million years ago^2^), the evolution of shell-crushing (durophagous) and boring predation in marine organisms caused a change from the dominance of sedentary, epifaunal suspension feeders to more mobile organisms including infauna and predators^2–5^. It is thought that the MMR heavily affected the stalked crinoids (sea lilies), making the majority of forms extinct as their sessile nature made them easy prey for durophagous predators in shallow waters. Stalked isocrinid crinoids (Order Isocrinida) were displaced into deeper water^4,6–8^, potentially by the more mobile comatulid crinoids (featherstars, Order Comatulida), which were better able to evade predation and which underwent a series of radiations during the MMR^9,10^.

Today stalked isocrinids are almost entirely restricted to deeper water environments, their shallowest occurrences being 100–170 m in the western Pacific^11,12^ and western Atlantic^6,13^. They occur to depths of 200–300 m and, rarely, they occur at >400 m^14^. Isocrinids are more mobile than other stalked forms and capable of local relocation^15–18^. Despite this, it was thought that isocrinids were restricted to middle-shelf and deeper environments during the Late Cretaceous and to outer-shelf and deeper by the Eocene^6,13^.

There is fossil evidence for an increase in predation on shallow water crinoids in the Mesozoic^1,10^, including an increased frequency of bite marks and rate of regenerated arms as a result of autotomy (arm shedding)^12,19^. In modern populations, elevated rates of predation in shallower waters compared with deep waters has also been cited as evidence of increased predation during the MMR^12,19^. However, the main lines of evidence for changes in predation intensity on isocrinids bought about by the MMR are the apparent lack of isocrinids from shallow water fossil sites in the Cenozoic and their absence from shallow waters at the present day.

Globally, the fossil record of stalked crinoids is extremely good for the Middle to Late Cretaceous^20–22^. Deep water isocrinid occurrences are found from the early Eocene (Rösnäs Formation, Denmark^20^, the Eocene London Clay, England^23^), the early Oligocene (Keasey Formation, Oregon, USA^24–27^), the late Oligocene (West Indies^28^), the Miocene (Japan^29,30^) and the Pliocene (Philippines^31^), and these are consistent with the argument that stalked crinoids migrated from shallower to deeper water in the early Cenozoic^4,6–8^. However, in the Northern Hemisphere some shallow water isocrinids persisted until the end of the Danian^20,24^, and there are a few isolated occurrences from the late Paleocene^6^ and the late Oligocene^6^. Recently stalked crinoids have been described from the early Paleogene of Central Europe^21^, indicating that stalked forms remained in shallow water settings for some time after the initiation of the MMR, until the late Mesozoic and into the early Cenozoic^21^. This led to the suggestion that predation intensity during the Mesozoic was not the only factor controlling the presence or absence of stalked forms in shallow and deep water environments^22^ and the off-shore displacement of isocrinids was a gradual process that occurred later than previously supposed^9^. Isolated occurrences of Cenozoic stalked isocrinids from Antarctica^32–36^, New Zealand^37–46^, South America^47^, and Australia^48^, have also been described from shallow water deposits. Explanations for the South American and Antarctic occurrences have focused on a hypothetical reversion to Palaeozoic type communities in response to environmental perturbations^35,47,49^. However, isolated occurrences of isocrinids in the Cenozoic have led to suggestions that the MMR was not globally synchronous^9,22,34^ or that there was a possible delayed onset of MMR^38^ in Southern Hemisphere regions.

We describe 37 new Antarctic and Australian isocrinid occurrences of isolated columnals (often ignored in evolutionary studies) and articulated crowns, assigned to nine different species in three genera. Crinoids from the Cenozoic basins of Australia, one of the largest packages of shallow water sediment of this age, have not been studied in detail and, until now, have only yielded one crinoid occurrence^48^. Exhaustive studies of museum collections and detailed provenance information were applied together with field sampling. Antarctic isocrinids were collected with detailed sedimentological information, enabling accurate environmental and temporal placement. In addition to previously described fossil occurrences^32–48^, this substantial new body of data indicates that the Southern Hemisphere was an important shallow water isocrinid province during the Paleogene. The data presented herein provides conclusive evidence that the migration of stalked isocrinids from shallow to deep water did not occur at the same time all over the world.

## Results

### Identification of new isocrinid species

**Fig. 1:**
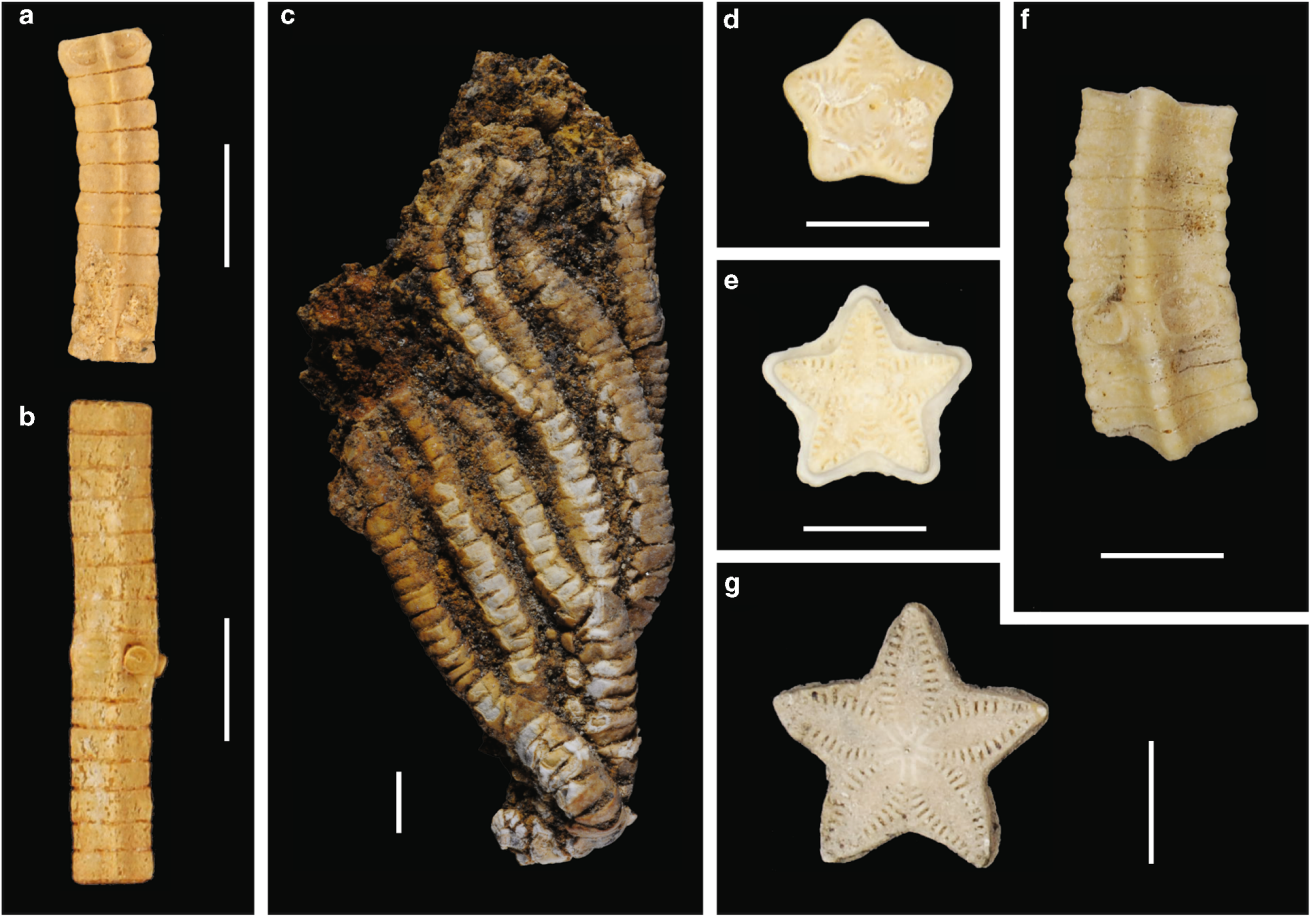
Examples of newly discovered and described Southern Hemisphere stalked crinoids. **a**, **b** *Isocrinus* sp. 1 lateral surface views (**a** WAM 88.32; **b** WAM 88.6) Cardabia Formation (Wadera Calcarenite Member), Paleocene, Western Australia. **c** *Saracrinus* sp. lateral side of the crown (D.916.1) from the Cross Valley Formation, Seymour Island, Antarctica. **d**, **e** *Metacrinus* sp. 2 articular surface views (‘Katie’s Stars’ WAM 17.1938) from Nanarup Limestone, middle Eocene, Western Australia. **f** *Metacrinus* sp. 2 lateral surface views (WAM 88.374a) Wilson Bluff Limestone (Toolina Limestone) middle Eocene, Western Australia. **g** *Metacrinus* sp. 3 articular surface views (WAM 17.1937) Wilson Bluff Limestone (Toolina Limestone) middle Eocene, Western Australia. Scale bars = 5 mm

**Fig. 2:**
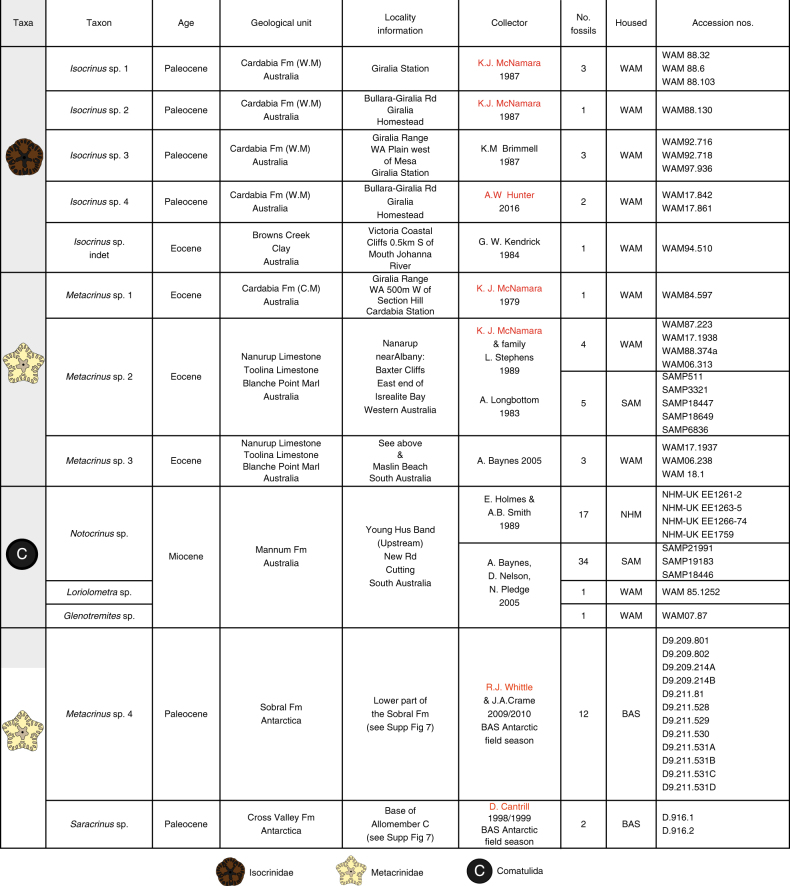
Information for newly identified fossils included in this study. Descriptions and images of these specimens can be found in the Supplementary Note 1 and Supplementary Figures 1–5. Names in red indicate authors on this paper who originally collected a large proportion of the material in the field. Materials collected by other people, undescribed before this study, were accessed through the institutions in which they are housed. WM Wadera Member, CM Cashin Member, WAM Western Australian Museum, SAM South Australian Museum, BAS British Antarctic Survey, NHM Natural History Museum, UK

**Fig. 3:**
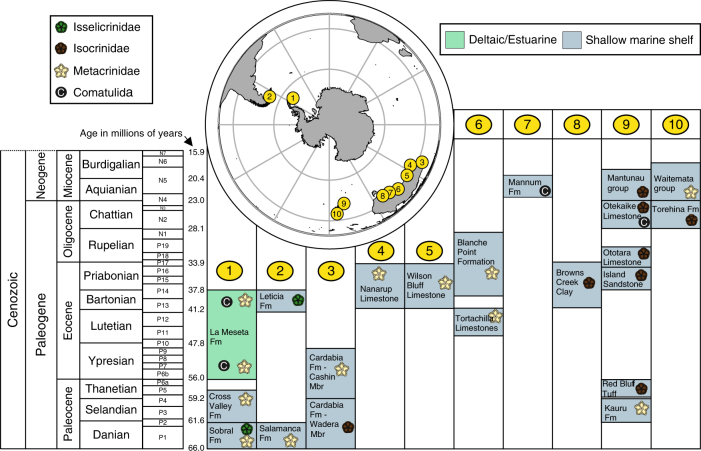
Distribution of shallow marine stalked crinoids in the Cenozoic of the Southern Hemisphere. Newly discovered and described fossils along with those previously described from Antarctica^32–36^, New Zealand^37–46^, South America^47^, and Australia^48^ are shown in the geological units they were found in (foram zones from McGowran^87^ are given next to geological stages). Numbered localities are: 1. Antarctic Peninsula – Seymour Island, 2. South America – Patagonia, 3. Western Australia–Carnarvon Basin, 4. Western Australia, western Eucla Basin, 5. Western Australia–eastern Eucla Basin, 6. South Australia Murray Basin. 8. South Australia–Otway Basin, 9. New Zealand South Island, 10. New Zealand, North Island. Map outline modified from Seton et al.^88^ Geological settings were identified as being shallow water (inner shelf or shallower) based on field studies and published literature (Supplementary Note 2), taxonomic descriptions of the new taxa are detailed in Supplementary Note 1

**Fig. 4:**
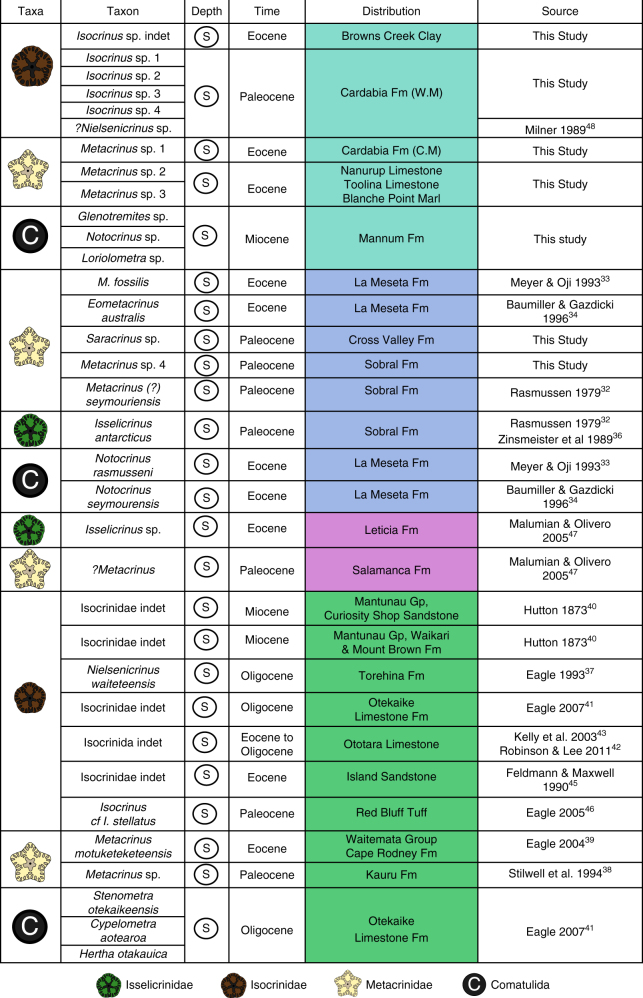
Distribution data for taxa mentioned in Fig. 3, with data sources for this information. All samples were collected in shallow water. In the Distribution column Australian localities are presented in light blue, Antarctic localities are displayed in dark blue, South American localities are shown in pink, New Zealand localities are presented in green

Nine new Cenozoic species (and one indeterminate species) of the Order Isocrinida are identified from shallow water deposits in Antarctica and Australia (Figs. 1and 2) using traditional crown characters as well as columnals or sets of columnals (pluricolumnals) (Supplementary Note 1, Supplementary Figs. 1–5). Three genera of the Order Comatulida are identified from Australia (Fig. 2, Supplementary Note 1, Supplementary Fig. 5c, g-i). Two different isocrinid families are identified: the Metacrinidae (*Metacrinus* and *Saracrinus*) and Isocrindae (*Isocrinus*). New occurrences of Metacrinidae are identified from Antarctica (Figs. 1–4, Supplementary Note 1, Supplementary Figs. 4 and 6); and Metacrinidae plus Isocrinidae from Australia (Figs. 1–4, Supplementary Note 1, Supplementary Figs. 1–3, 5 and 7). A taxonomic monograph describing all of these new species is in production.

### Western Australian isocrinids

**Fig. 5:**
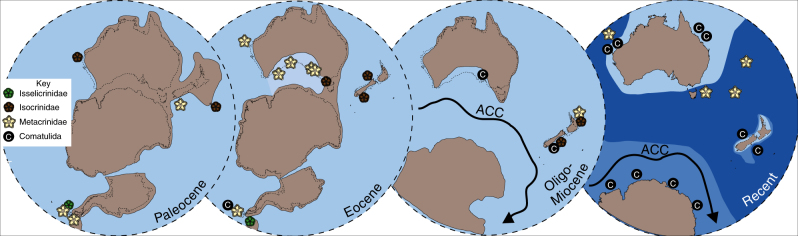
Cenozoic Southern Hemisphere crinoid distribution. The distribution of the Order Isocrinida (Families Isselicrinidae, Isocrinidae and Metacrindae) and Order Comatulida in the Cenozoic of the Southern Hemisphere, from the Paleocene to the Recent. Map outlines modified from Seton et al.^88^. Darker blues indicate deeper water

In Western Australia four Paleocene species of *Isocrinus* are identified from shallow marine shelf strata in the Carnarvon Basin (Figs. 2–5, Supplementary Notes 1 and 2, Supplementary Figs. 1, 2 and 7), and isocrinids persisted in this region until the Eocene (*Metacrinus* sp. 1). *Metacrinus* species are identified from shallow water deposits in Western and Southern Australia, from the middle and late Eocene (*Metacrinus* sp.1, Carnarvon Basin, *Metacrinus* sp. 2; Eucla and St Vincent Basin, *Metacrinus* sp. 3 Eucla and St Vincent Basin) (Fig. 2, Supplementary Figs. 3, 5d – f and 7 and Supplementary Notes 1 and 2). An indeterminate species of *Isocrinus* is identified from Eocene shallow water sediments of the Otway Basin, Victoria (Figs. 2–4, Supplementary Fig. 5a, b and Supplementary Notes 1 and 2). In Australia comatulids (the following genera are identified: *Glenotremites* sp., *Notocrinus* sp., and *Loriolometra* sp., Figs. 2–5, Supplementary Note 1, Supplementary Fig. 5c, g–i) first appear in the fossil record in the early Miocene shallow water Mannum Formation^50^ (Supplementary Note 2). Our descriptions (Supplementary Note 1) of previously collected specimens represent the richest accumulation of fossil comatulids in the Southern Hemisphere.

### Antarctic isocrinids

New specimens of *Metacrinus* are identified from Antarctic Paleocene deltaic sediments on Seymour Island (*Metacrinus* sp. 4, Sobral Formation, Supplementary Figs. 4 and 6, Supplementary Notes 1 and 2). These are the oldest confirmed specimens of *Metacrinus* in the fossil record. Previously described Maastrichtian specimens^32^ have been cited as being identified from the Sobral Fm and are thus probably also Paleocene in age (Figs. 3 and 4, Supplementary Note 2). *Saracrinus* sp., also identified from Seymour Island, inhabited a very shallow marine environment (Cross Valley Formation, Supplementary Note 2). This is the oldest confirmed occurrence of the extant genus *Saracrinus* in the fossil record (Supplementary Note 1). Several Cretaceous and Eocene occurrences of isocrinids have already been described from Seymour Island^32–36^, and fossil comatulids have previously been described from Antarctica from the early^34^ and late Eocene^33^ (Figs. 3, 4).

In addition to previously published shallow water Cenozoic Australian^48^, New Zealand^37–46^, Patagonian^47^ and Antarctic specimens^32–36^ (Fig. 4), our new occurrences (Fig. 2) provide evidence for a Southern Hemisphere Paleocene to Eocene faunal province inhabited by shallow water isocrinids (Figs. 3 and 5). Isocrinids were not present above the Eocene/Oligocene boundary in Australia or Antarctica; but remained in New Zealand shallow waters until the early Miocene^39^ (Figs. 3 and 5). The more motile comatulids first occur in the fossil record of Antarctica in the early Eocene and appeared in abundance in Australia in the early Miocene (Figs. 3 and 5).

## Discussion

The nine newly identified Cenozoic Southern Hemisphere isocrinid species (Fig. 2), and previously identified occurrences^32–48^ which have been compiled together for the first time to our knowledge (Fig. 4), confirm that the response of stalked crinoids to increased predation pressure as part of the MMR was asynchronous^34,38^. Our data refute the hypothesis that the Antarctic and South American benthic communities experienced periodic reversions to a Palaeozoic type community structure as a response to environmental perturbations^35,47^. The new data provided herein, in addition to previously published occurrences^32–48^, demonstrate that a shallow water Southern Hemisphere fauna of isocrinid crinoids persisted over the Cretaceous-Paleogene boundary, continued into the early Paleocene and to at least the Eocene/Oligocene boundary (Figs. 3 and 5). The shift in distribution of isocrinids out of shallow water may have occurred at the end of the Eocene around Antarctica and Australia, and later in the Miocene in New Zealand. The modern deep water Isocrinida *Metacrinus* and *Saracrinus* may have evolved from shallow water Antarctic habitats in the Paleocene, spreading to the southern margin of Australia in the Eocene, and to their present distribution in deeper waters around Australia, New Zealand, New Caledonia, Indonesia, the Philippines and Japan^14,51–53^.

The late persistence of isocrinid crinoids in Antarctica, Australia, New Zealand and South America could be explained either as a result of an absence of, or reduced durophagous predation during the MMR in the Southern Hemisphere. Alternatively, it could be as a result of a delayed distribution and/or radiation of motile and more competitive comatulid crinoids which had greater success in shallow waters than the less mobile isocrinids^13^. These two options are considered below.

The role of durophagous predation in relation to the distribution of isocrinid crinoids is difficult to assess because, until recently, there was little information about predation on crinoids^1,10,54–56^. Diving investigations have shown predation on recent comatulid crinoids by fishes of several families, consisting of sublethal damage to the crinoid visceral mass and arms^56^. Crinoid ossicles from the Order Millericrinida were found in bromalites from the Triassic; durophagous sharks, colobodontid fish, placodonts, and some pachypleurosaurs or sauropterygian reptiles were suggested as possible predators^57^. Predation on comatulid crinoids by cidaroid echinoids has been indicated by studying bite marks on crinoid columnals as well as through direct observation^1,10^. However, thus far, the only confirmed evidence of predation on isocrinid crinoids has come from laboratory observations and in situ observations using submersibles of predation by cidaroid echinoids^10^. Therefore, echinoid predation was suggested as a major driver of crinoid radiation and diversity in the Mesozoic^1,10^. Predation has also been inferred by looking at arm loss and regeneration, suggested to be a response to predation, in fossil isocrinids like *Metacrinus* from the La Meseta Formation^33^.

Latitudinal differences in predation may explain the patterns of Cenozoic isocrinid depth distribution seen in the Southern Hemisphere, if predation pressure decreased with increasing latitude^3^. In modern brachiopods, lower frequencies of repaired predator attacks were observed at high latitudes, possibly due to a lower diversity of crushing predators^58^. However, it is only today that durophagous predators are rare or absent from Antarctica^59^. The presence of isocrinids in the La Meseta Formation was attributed to the population being subjected to lower predation pressure than generally prevailed in post-Mesozoic shallow water environments^33^ as the isocrinids had a lower rate of regenerated arms than in modern settings^33^. However, taxa thought to predate upon crinoids are found along with isocrinids in Antarctic deposits so a lack of predators cannot be invoked to explain the presence of the isocrinids in the region at the time. Teleost fish, crustaceans and sharks are found in Cretaceous, Paleocene and Eocene deposits of Antarctica^60–64^ in the same formations as isocrinids. The same is true for Western Australian Eocene deposits (K. McNamara pers. obser.). Isocrinids also co-occur with spines of cidaroid echinoids (known to predate on isocrinids^10^) in the Sobral Formation, and cidaroid echinoids have also been described from the La Meseta Formation^65^. Similarly cidaroids and isocrinids are both common in the middle Eocene Nanarup Formation in south-western Australia (McNamara pers. obser.).

Isocrinids are capable, as are comatulids, of autotomy to avoid predatory attacks^15^. Autotomy planes in stalks and arms and muscular articulations allowing rapid crawling originated in the Middle Triassic^57^. This, along with recent evidence that isocrinids are motile^15^, indicates that isocrinids evolved adaptations that enabled them to evade predators during the Mesozoic. Recent specimens of the isocrinids *Metacrinus, Saracrinus* and *Endoxocrinus* have been shown to exhibit arm regeneration^12,19^. *Endoxocrinus* shows a greater frequency of arm regeneration in shallower (~150 m deep) water than in deeper water (~750 m), leading to the suggestion that predation in shallow water caused isocrinids to move to deeper water^12^. However, this also shows that today isocrinids are able to inhabit areas which are subject to predation. Isocrinids have been subject to predation throughout their evolutionary history, and have evolved strategies to deal with predatory attacks. Salamon and Gorzelak^22^ suggested that predation intensity during the Mesozoic was not the only factor controlling the presence or absence of stalked forms in shallow and deep water environments and our data seem to be consistent with this.

Comatulids (feather stars) are thought to have had a higher survival capacity in shallow water than stalked isocrinids^13^ due to their greater adaptability^13^. This resulted in comatulids becoming dominant in shallow waters at the present day^66^. The timing of the onset of comatulid radiation may have not been globally consistent, accounting for longer survival for isocrinids in shallow waters in the Southern Hemisphere. The first true comatulids date from the Early Jurassic^66^, but overall their fossil record is poor due to a lack of articulated fossils. Using disarticulated elements relies heavily on finding a single centrodorsal ossicle, as arm ossicles are largely taxonomically indeterminate. The oldest known Antarctic comatulid (*Notocrinus*) was described from the early Eocene and co-occurred with isocrinids^34^. In South Australia, specimens of comatulids (*Glenotremites, Notocrinus*, and *Loriolometra*–Notocrinidae) have been collected in abundance^50^ from the shallow water early Miocene Mannum Formation, with no co-occurring Isocrinida. This may indicate comatulid dominance in the marine community.

Here we show that Australia has a shallow water fossil record of Isocrinida from the Paleocene to the end of the Eocene (Fig. 3). The oldest (Paleocene) Australian Isocrinida are from Western Australia (Fig. 3). At this time the southern margin of Australia was still connected to Antarctica^67^ (Fig. 5), but a transgression in the north led to the formation of a shallow water basin^68^, which the Isocrinida inhabited until the early Eocene. Australia finally separated from Antarctica later in the Eocene, forming an embayment with a complex of shallow water basins from west to east across the southern margin of the Australian continent (Fig. 5). Like echinoids^69^, foraminifera^70^, and brachiopods^71^, the Isocrinida show a pattern of dispersal in a southerly direction along the western Australia coast during the early Paleogene, then an easterly spread across the southern margin of the Australian continent (Fig. 5). Isocrinids do not occur in post- Eocene strata in Australia (Figs. 3 and 5), having seemingly been replaced by comatulids in shallow water habitats. New Zealand was left as an apparent shallow water refugium for isocrinids until the early Miocene (Fig. 3), isocrinids having persisted here from the Paleocene (Figs. 3 and 5)^37–46^. Following this, isocrinids were displaced to deeper water environments, which they still inhabit today^14^.

Isocrinids inhabited Antarctic shallow water communities until the end of the Eocene^33^ (Fig. 3). There is no evidence for fossil isocrinids in Antarctica, Australia or South America after the Eocene (Figs. 2 and 4). This was a time of speciation and radiation in the Southern Hemisphere for many taxa, including comatulids^72,73^ when changes in continental configuration and ocean circulation brought in different water masses and isolated Antarctic marine faunas^74^. The Antarctic Circumpolar Current (ACC) started around the Eocene⁄Oligocene boundary to early Oligocene^75^ physically isolating Antarctica and preventing warmer water masses from reaching the continent. Full development of the ACC resulted in faunal turnover in the Southern Hemisphere, and an increase in cool water cosmopolitan and true Antarctic endemic forms^76,77^. This is supported by molecular clock data, which shows that modern species of the comatulid *Promachocrinus* evolved in the Antarctic region after the onset of the ACC^73^. Similar radiation events after the onset of the ACC are seen in other taxa such as amphipods, isopods and octopods^72^. The radiation of apparently more successful modern comatulid taxa in the Southern Hemisphere is co-incident with the demise of isocrinids in the region. The onset of the ACC may have caused a local extinction of isocrinids in the Southern Ocean. The repeated extension of ice sheets across the Antarctic continental shelf may also have discouraged the less mobile isocrinids from living at the depths at which they are found elsewhere today.

Overall, based on the evidence presented herein, it is clear that isocrinids inhabited shallow waters in the Southern Hemisphere region in the early Cenozoic, with the oldest metacrinid specimens found in Antarctica. Opening seaways resulted in isocrinids dispersing along newly formed shallow Australian basins around the southern margin of Australia to New Zealand.

## Methods

### Taxonomic study of isocrinids

The taxonomy of Cenozoic crinoids is virtually unstudied^24^ other than the notable exceptional occurrences where the crowns have been preserved such as the Rösnäs Formation (Eocene), Denmark, the London Clay (Eocene), England, the Keasey Formation (Oligocene) Mist, Columbia County, Oregon and the La Meseta Formation (Eocene), Seymour Island, Antarctica. The vast majority of material consists of single columnals or sets of columnals, much of which is in need of revision^24^. We used a new systematic framework based on recent taxonomic work on Jurassic and Cretaceous^65^ taxa and applied this to the new taxa collected from Australia and Antarctica (Supplementary Note 1). We also compared specimens to recent isocrinids from the Natural History Museum (NHM) UK and the University of Tokyo Museum. Articulated isocrinid crinoids are typically identified based on the number of brachials in the arms and their surface ornamentation. The systematics of isocrinid crinoids has been previously restricted to characters within the crown. In contrast, taxonomy using stem columnals or sets of columnals (pluricolumnals) is considered problematic^78^. However, there are studies which have extensively utilised columnals in the absence of preserved cup material^79–81^. We use the methodology detailed in these studies and summarised in Supplementary Fig. 8 for the material described herein. Taxonomic features include the outer surface of the stem (latera), the shape and articular face of the columnals, and its articulations (Supplementary Fig. 8). Sets of columnals called pluricolumnals typically represent stem segments shed in life. These can be quickly incorporated into the sediment or can remain in the substrate where they are subject to abrasion or local transport. The majority of the columnals have not been abraded, suggesting little transport^81,82^; the high number of articulated sets of columnals in the dataset also suggests rapid burial of columnal segments. However, it should be noted that articulated stalks and headless erect stalks have been observed to survive in the deep-sea and in lab-held *Metacrinus* from Japan^83^. Therefore, some caution is needed in claiming that articulated lengths of stalk found widely in the fossil record indicate rapid burial.

### Sample collection

Information about the collecting localities of the newly identified specimens in this study can be found in Fig. 2, Supplementary Figs. 6 and 7, and Supplementary Notes 1 and 2. Twelve specimens of *Metacrinus* sp. 4 from the Paleocene Sobral Formation Seymour Island, Antarctica, were collected in the 2009/2010 British Antarctic Survey (BAS) field season. These fossils were collected by R.J. Whittle and J.A. Crame in conjunction with section lines measured using an Abney level and Jacobs staff, along with detailed field studies and sedimentological logging by J. Francis and J. Ineson (Fig. 2, Supplementary Figs. 4c, d and 6). They are preserved as pluricolumnals only. Two specimens of *Saracrinus* from the Cross Valley Formation, Seymour Island, Antarctica, were collected by David Cantrill in the 1998/1999 BAS field season (Fig. 2, Supplementary Figs. 4a, b and 6). They are very well preserved with arms attached to the calyx, but with no stalk. To aid identification of Antarctic material, taxonomic comparisons were made with Seymour Island fossil specimens in collections at the Springer Room, National Museum of Natural History, Smithsonian Institution, Washington DC and with modern taxa at the Natural History Museum, London. The ages for the sections and the specimens collected were based on Bowman et al.^84^. Data for water depth for Antarctic localities was based on the field studies of Dr J. Ineson (Geological Survey of Denmark and Greenland) and have also been the focus of geological study from other authors^85,86^.

Geological settings and environment of deposition including water depths for rock units mentioned herein are given in Supplementary Note 2 along with the supporting literature references for their interpretation. Herein shallow water is defined as occurring on the inner shelf or shallower.

The 23 Australian crinoid specimens came from spot sampling in the field and museum collections; previously overlooked data from disarticulated columnals were also included. The Paleocene Australian specimens were sampled by A.W. Hunter from the Cardabia Formation (Giralia Anticline, north part of the Southern Carnarvon Basin, Supplementary Fig. 7). Paleocene to Oligocene Australian data came from the series of basins that form the Great Bight Basin System (Supplementary Note 2, Supplementary Fig. 7) and the Southern Carnarvon Basin. They were sampled over a 30 year period by K.J. McNamara and team (S.P. Radford, K.A. McNamara, T. McNamara, J. McNamara, A. Baynes, K.M. Brimmell, G.W. Kendrick and A. Longbottom). Comatulid specimens from the Mannum Formation were collected by A. Baynes, D. Nelson, N. Pledge, E. Holmes and A.B. Smith. To aid identification of the Australian specimens, extant material was studied in reference collections in the Muséum National d’Histoire Naturelle, Paris, the Natural History Museum, London (NHM), the Western Australian Museum, Perth (WAM), the Southern Australian Museum, Adelaide (SAM), the Museum of Victoria, the Australian Museum, Sydney, the University of Tokyo Museum, and the National Museum of Natural History, Smithsonian Institution. Monographs of Cenozoic taxa plus specimens and monographs of modern taxa were compared. Australian and Antarctic fossil sample data were combined with published data from Australia, Antarctica, South America and New Zealand.

### Data availability

Information regarding the data that support the findings of this study are available within the paper, Supplementary Figures and Supplementary Notes 1 and 2. All Antarctic fossil specimens are deposited at the British Antarctic Survey, Cambridge. Australian specimens are housed in the Western Australian Museum, South Australian Museum and the Natural History Museum (UK). Detailed provenance information for the newly collected specimens is given in Fig. 2, and Supplementary Notes 1 and 2.

### Accession Numbers

Western Australian Museum-WAM 88.32, WAM 88.6, WAM 88.103, WAM 88.130, WAM 92.716, WAM 92.718, WAM 97.936, WAM 17.842, WAM 17.861, WAM 94.510, WAM 84.597, WAM 87.223, WAM 17.1938, WAM 88.374a, WAM 06.313, WAM 17.1937, WAM 06.238, WAM 18.1, WAM 85.1252 and WAM 07.87.

South Australian Museum-SAM P511, SAM P3321, SAM P18447, SAM P18649, SAM P6836, SAM P21991, SAM P19183 and SAM P18446.

Natural History Museum (UK)-NHM-UK EE 1261-2, NHM-UK EE 1263-5, NHM-UK EE 1266-74 and NHM-UK EE 1759.

British Antarctic Survey (Cambridge)-D9.209.801, D9.209.802, D9.209.214 A, D9.209.214B, D9.211.81, D9.211.528, D9.211.529, D9.211.530, D9.211.531 A, D9.211.531B, D9.211.531 C, D9.211.531D, D.916.1 and D.916.2.

## Electronic supplementary material

Supplementary Information

## References

[1] Gorzelak P, Salamon MA, Baumiller TK (2012). Predator-induced macroevolutionary trends in Mesozoic crinoids. Proc. Natl Acad. Sci. USA.

[2] Vermeij GJ (1977). The Mesozoic Marine Revolution: evidence from snails, predators and grazers. Paleobiology.

[3] Vermeij GJ (1987). Evolution and Escalation: An Ecological History of Life.

[4] Harper, E. M. in *Predator-Prey Interactions in the Fossil Record* (eds Kelley, P. H., Kowalewski, M. & Hansen, T. A.) pp. 433–455, (Kluwer Academic/Plenum Publishers, New York, 2003).

[5] Wagner PJ, Kosnik MA, Lidgard S (2006). Abundance distributions imply elevated complexity of Post-Paleozoic marine ecosystems. Science.

[6] Bottjer DJ, Jablonski D (1988). Paleoenvironmental patterns in the evolution of post-Paleozoic benthic marine invertebrates. Palaios.

[7] Aronson RB (1994). Scale-independent biological interactions in the marine environment. Oceanogr. Mar. Biol. - Annu. Rev..

[8] Jablonski D, Sepkoski JJ (1996). Paleobiology, community ecology, and scales of ecological pattern. Ecology.

[9] Gorzelak P, Salamon MA, Trzęsiok D, Lach R, Baumiller TK (2016). Diversity dynamics of post-Palaeozoic crinoids – in quest of the factors affecting crinoid macroevolution. Lethaia.

[10] Baumiller TK (2010). Post-Paleozoic crinoid radiation in response to benthic predation preceded the Mesozoic marine revolution. Proc. Natl Acad. Sci. USA.

[11] Oji, T. in *Current Aspects of Biogeography in West Pacific and East Asian Regions. Nature and Culture, No. 1*., (eds Ohba, H., Hayami, I., Mochizuki, K.) pp. 27–43, (The University Museum, The University of Tokyo, 1989).

[12] Oji T (1996). Is predation intensity reduced with increasing depth? Evidence from the West Atlantic Stalked Crinoid *Endoxocrinus parrae* (Gervais) and Implications for the Mesozoic Marine Revolution. Paleobiology.

[13] Meyer DL, Macurda DB (1977). Adaptive radiation of comatulid crinoids. Paleobiology.

[14] Ameziane-Cominardi N (1991). Distribution bathymetrique des pentacrines du Pacifique occidental: essai de modelisation et d’application aux faunes du lias: problèmes de tectono-eustatisme au cours du rifting téthysien. Doc. Des. Lab. De. géologie Lyon.

[15] Baumiller TK (2008). Crinoid Ecological Morphology. Annu Rev. Earth Planet Sci..

[16] Baumiller TK, Messing CG (2007). Stalked crinoid locomotion, and its ecological and evolutionary implications. Palaeontol. Electron..

[17] Baumiller TK, LaBarbera M, Woodley JD (1991). Ecology and functional morphology of the isocrinid *Cenocrinus asterius* (Linnaeus) (Echinodermata: Crinoidea): *in situ* and laboratory experiments and observations. Bull. Mar. Sci..

[18] Messing CG, RoseSmyth MC, Mailer SR, Miller JE (1988). Relocation movement in a stalked crinoid (Echinodermata). Bull. Mar. Sci..

[19] Oji T (1986). Skeletal variation related to arm regeneration in *Metacrinus* and *Saracrinus*, Recent stalked crinoids. Lethaia.

[20] Rasmussen HW (1972). Lower Tertiary Crinoidea, Asteroidea and Ophiuroidea from Northern Europe and Greenland. Det. K. Dan. Vidensk. Selsk. Biol. Skr..

[21] Salamon MA, Gorzelak P, Borszcz T, Gajerski A, Kaźmierczak J (2009). A crinoid concentration Lagerstätte in the Turonian (Late Cretaceous) *Conulus* Bed (Miechów-Wolbrom area, Poland). Geobios.

[22] Salamon M, Gorzelak P (2010). Late Cretaceous crinoids (Crinoidea) from Eastern Poland. Palaeontogr. Abt. A.

[23] Wignall PB, Simms MJ (1990). Pseudoplankton. Palaeontology.

[24] Hess, H. in *Fossil Crinoid*s (eds Hess, H., Ausich, W. I., Brett, C. E. & Simms, M. J.) pp. 233–236 (Cambridge University Press, Cambridge, UK, 1999).

[25] Moore RC, Vokes HE (1953). Lower Tertiary Crinoids from Northwestern Oregon. Geol. Surv. Prof. Pap..

[26] Burns, C. & Mooi, R. in *From Greenhouse to Icehouse: The Marine Eocene-Oligocene Transition* (eds Prothero, D. R., Ivany, L. C. & Nesbitt, E. A.) pp. 88–106, (Columbia University Press, New York, 2003).

[27] Burns C, Campbell KA, Mooi R (2005). Exceptional crinoid occurrences and associated carbonates of the Keasey Formation (Early Oligocene) at Mist, Oregon, USA. Palaeogeogr. Palaeoclimatol. Palaeoecol..

[28] Donovan SK, Harper DAT, Portell RW (2015). In deep water: a crinoid–brachiopod association in the Upper Oligocene of Antigua, West Indies. Lethaia.

[29] Oji T (1990). Miocene Isocrinidae (stalked Crinoids) from Japan and their biogeographic implication. Trans. Proc. Palaeontol. Soc., Jpn. N. S..

[30] Fujiwara SndashI, Oji T, Tanaka Y, Kondo Y (2005). Relay Strategy and adaptation to a muddy environment in *Isselicrinus* (Isselicrinidae: Crinoidea). Palaios.

[31] Donovan SK, Helwerda RA (2016). Neogene crinoids of southeast Asia: preservation, systematics and significance. Alcheringa.

[32] Rasmussen HW (1979). Crinoideos del Cretacico superior y del Terciario inferior de la Isla Vicecomodoro Marambio (Seymour Island), Antartida. Contrib. Científico Del. Inst. Antártico Argent..

[33] Meyer DL, Oji T (1993). Crinoids from Seymour Island, Antarctic Peninsula: Paleobiogeographic and Paleoecologic implications. J. Paleontol..

[34] Baumiller TK, Gaździcki A (1996). New crinoids from the Eocene La Meseta Formation of Seymour Island, Antarctic Peninsula. Palaeontol. Pol..

[35] Aronson RB, Blake DB, Oji T (1997). Retrograde community structure in the late Eocene of Antarctica. Geology.

[36] Zinsmeister WJ, Feldmann RM, Woodburne MO, Elliot DH (1989). Latest Cretaceous/Earliest Tertiary transition on Seymour Island, Antarctica. J. Paleontol..

[37] Eagle MK (1993). A new fossil isocrinid crinoid from the Late Oligocene of Waitete Bay, Northern Coromandel. Rec. Auckl. Inst. Mus..

[38] Stilwell JD, Fordyce RE, Rolfe PJ (1994). Paleocene Isocrinids (Echinodermata:Crinoidea) from the Kauru Formation, South Island, New Zealand. J. Paleontol..

[39] Eagle MK (2004). *Saracrinus* (Crinoidea: Metacrininae) from the Early Miocene of Motuketekete Island, Hauraki Gulf, Auckland, New Zealand. Rec. Auckl. Mus..

[40] Hutton, F. W. *Catalogue of the Tertiary Mollusca and Echinodermata of New Zealand in **The Collection of the Colonial Museum* (New Zealand Geological Survey, Wellington, 1873; 48).

[41] Eagle MK (2007). New Fossil Crinoids (Articulata: Comatulida) from the Late Oligocene of The Pentland Hills and Hurstlea, South Island. Rec. Auckl. Mus..

[42] Robinson JH, Lee DE (2011). A shallow, warm-water calcitic molluscan fauna from an Early Oligocene seamount, North Otago, New Zealand. N.Z. J. Geology Geophysics.

[43] Kelly M, Lee D, Kelly S, Buckeridge JS (2003). A recent sponge, Pleroma aotea Kelly (“Order” Lithistida: Family Pleromidae), in the late Eocene Ototara Limestone of Otago, New Zealand. N.Z. J. Mar. Freshw. Res..

[44] Campbell HJ (1993). Cretaceous-Cenozoic geology and biostratigraphy of the Chatham Islands, New Zealand. Monogr. Inst. Geol. Nucl. Sci..

[45] Feldmann RM, Maxwell PA (1990). Late Eocene decapod Crustacea from North Westland, South Island, New Zealand. J. Paleontol..

[46] Eagle MK (2005). A new genus of fossil crinoid (Cyrtocrinidia: Sclerocrinidae) from Chatham Island, New Zealand. Rec. Auckl. Mus..

[47] Malumián N, Olivero EB (2005). Shallow-water late middle Eocene crinoids from Tierra del Fuego: a new southern record of a retrograde community structure. Sci. Mar..

[48] Milner GJ (1989). The first record of an isocrinid crinoid from the Tertiary of Australia. Rec. West. Aust. Mus..

[49] Aronson RB, Blake DB (2001). Global climate change and the origin of modern Benthic communities in Antarctica. Am. Zool..

[50] Lukasik JJ, James NP (1998). Lithostratigraphic revision and correlation of the Oligo‐Miocene Murray Supergroup, Western Murray Basin, South Australia. Aust. J. Earth Sci..

[51] Oji T, Kitazawa K (2008). Discovery of two rare species of stalked crinoids from Okinawa Trough, southwestern Japan, and their systematic and biogeographic implications. Zool. Sci..

[52] Oji T, Kitazawa K (2006). Distribution of stalked crinoids (Echinodermata) from waters off the southern coasts of Japan. Mem. Natl. Sci. Mus., Tokyo.

[53] Messing, C. *Metacrinus* Carpenter, 1882 in Messing, C. (2015) World List of Crinoidea. Accessed through: World Register of Marine Species at http://www.marinespecies.org/aphia.php?p=taxdetailsid=411397 on2016-03-01 (2015)

[54] Meyer, D. L. & Ausich, W. I. in* Biotic Interactions in Recent and Fossil Benthic* Communities (eds Tevesz, J. S. et al.) pp. 377–427, (Springer Science + Business Media, New York, 1983).

[55] Baumiller TK, Gahn FJ (2004). Testing predator-driven evolution with Paleozoic Crinoid arm regeneration. Science.

[56] Meyer DL (1985). Evolutionary implications of predation on Recent comatulid crinoids from the Great Barrier Reef. Paleobiology.

[57] Salamon MA, Niedźwiedzki R, Gorzelak P, Lach R, Surmik D (2012). Bromalites from the Middle Triassic of Poland and the rise of the Mesozoic Marine Revolution. Palaeogeogr. Palaeoclimatol. Palaeoecol..

[58] Harper EM, Peck LS (2016). Latitudinal and depth gradients in marine predation pressure. Glob. Ecol. Biogeogr..

[59] Aronson RB (2007). Climate Change and the Invasibility of the Antarctic Benthos. Annu Rev. Ecol. Evol. Syst..

[60] Grande L, Chatterjee S (1987). New Cretaceous fish fossils from Seymour Island, Antarctic Peninsula. Palaeontology.

[61] Cione AL, Mercedes Azpelicueta de las M, Bellwood D (1994). An Oplegnathid fish from the Eocene of Antarctica. Palaeontology.

[62] Feldmann RM, Schweitzer CE (2006). Paleobiogeography of Southern Hemisphere decapod Crustacea. J. Paleontol..

[63] Griffiths HJ, Whittle RJ, Roberts SJ, Belchier M, Linse K (2013). Antarctic crabs: invasion or endurance. PLoS ONE.

[64] Whittle RJ, Quaglio F, Griffiths HJ, Linse K, Crame JA (2014). The Early Miocene Cape Melville Formation fossil assemblage and the evolution of modern Antarctic marine communities. Naturwissenschaften.

[65] Radwańska U (1996). A new echinoid from the Eocene La Meseta Formation of Seymour Island, Antarctic Peninsula. Palaeontol. Pol..

[66] Hess H (2014). Origin and radiation of the comatulids (Crinoidea) in the Jurassic. *Swiss*. J. Paleontol..

[67] Clarke JDA, Gammon PR, Hou B, Gallagher SJ (2003). Middle to Upper Eocene stratigraphic nomenclature and deposition in the Eucla Basin. Aust. J. Earth Sci..

[68] Hocking RM, Moors HT, Van de Graaff JE (1987). Geology of the Carnarvon Basin Western Australia. Geol. Surv. West. Aust. Bull..

[69] McNamara, K. J. in *Echinoderm Research 1998* (eds Candia Carnevali, M. D. & Bonasoro, F.) pp. 333–338, (A.A. Balkema, Rotterdam, 1999).

[70] McGowran B (2000). Australasian palaeobiogeography: the Palaeogene and Neogene record. Mem. Assoc. Australas. Palaeontol..

[71] Craig RS (2000). The Cenozoic Brachiopods of the Carnarvon Basin, Western Australia. Palaeontology.

[72] Newman L, Convey P, Gibson JAE, Linse K (2009). Antarctic Paleobiology: Glacial refugia and constraints on past ice-sheet reconstructions. PAGES News.

[73] Wilson NG, Hunter RL, Lockhart SJ, Halanych KM (2007). Multiple lineages and absence of panmixia in the “circumpolar” crinoid *Promachocrinus kerguelensis* from the Atlantic sector of Antarctica. Mar. Biol..

[74] Barnes DKA, Clarke A (2011). Antarctic marine biology. Curr. Biol..

[75] Barker PF, Filippelli GM, Florindo F, Martin EE, Scher HD (2007). Onset and role of the Antarctic circumpolar current. Deep Sea Res Part 2 Top. Stud. Oceanogr..

[76] Lazarus, D. & Caulet, J.–P. in *The Antarctic Paleoenvironment: A Perspective on Global Change Antarctic Research Series, 60* (eds Kennet, J. P. & Warnke, D. A.) pp. 145–174. (American Geophysical Union, Washington, DC, 1993).

[77] Brown B, Gaina G, Müller RD (2006). Circum-Antarctic palaeobathymetry: illustrated examples from Cenozoic to recent times. Palaeogeogr. Palaeoclimatol. Palaeoecol..

[78] Hess, H, Messing, C. G. & Ausich, W. I. in *Treatise on Invertebrate Paleontology, Part T, Echinodermata 2* (ed. Seldon, P.A.) pp. 1–261 (The University of Kansas Paleontological Institute, Lawrence, Kansas, 2011).

[79] Simms MJ (1989). British Lower Jurassic crinoids. Monogr. Palaeontogr. Soc., Lond..

[80] Hunter AW, Barras CG, Thuy B (2011). Online field-guide to fossils: British Middle Jurassic echinoderms. Proc. Geol. Assoc..

[81] Hunter AW, Underwood CJ (2009). Lithofacies and taphofacies control on distribution of crinoid habitats in the Bathonian (Middle Jurassic) of England and France. Acta Palaeontol. Pol..

[82] Hunter AW, Underwood CJ (2010). Comment and Reply on “Palaeoenvironmental Control on Distribution of Crinoids in the Bathonian (Middle Jurassic) of England and France” by Aaron W. Hunter and Charlie J. Underwood. Acta Palaeontol. Pol..

[83] Amemiya S, Oji T (1992). Regeneration in sea lilies. Nature.

[84] Bowman V (2016). The Paleocene of Antarctica: biostratigraphy and palaeogeographical implications for the palaeo-Pacific margin of Gondwana. Gondwana Res.

[85] Macellari CE (1988). Stratigraphy, sedimentology, and palaeoecology of Upper Cretaceous/Paleocene shelf-deltaic sediments of Seymour Island. Geol. Soc. Am. Mem..

[86] Marenssi S, Santillana S, Bauer M (2012). Estratigrafía, petrografía sedimentaria y procedencia de las formaciones Sobral y Cross Valley (Paleoceno), isla Marambio (Seymour), Antártica. Andean Geol..

[87] McGowran B (1979). The tertiary of Australia: foraminiferal overview. Mar. Micropaleontol..

[88] Seton M (2012). Global continental and ocean basin reconstructions since 200 Ma. Earth-Sci. Rev..

