# Supplementary material for: Globally discordant Isocrinida (Crinoidea) migration confirms asynchronous Marine Mesozoic Revolution
Source: Commun Biol. 2018 May 17;1:46. doi: 10.1038/s42003-018-0048-0 (PMC6123680; doi:10.1038/s42003-018-0048-0)
Supplement: Supplementary file 1 — Supplementary Information [file 42003_2018_48_MOESM1_ESM.pdf]

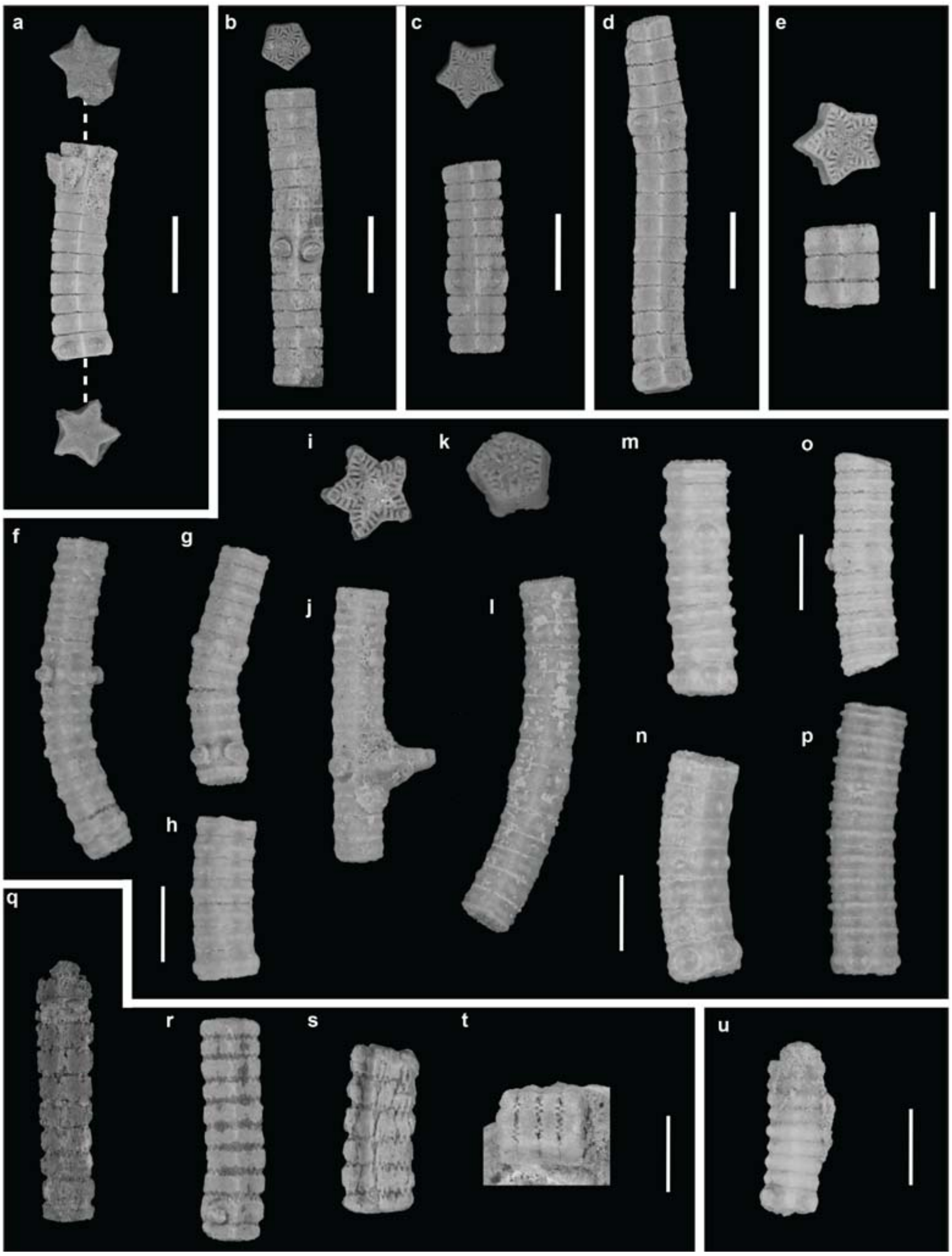

Supplementary Figure 1. New Australian taxa from the Cardabia Formation (Wadera Calcarenite Member), Paleocene, Western Australia. a-e articular surface views of, *Isocrinus* sp. 1 **a**, WAM 88.32. **b-c**, WAM 88.6. **d-e**, WAM 88.103. **f-p**, *Isocrinus* sp. 2, WAM 88.130. **q-t**, *Isocrinus* sp. 3. **q**, WAM 92.716. **r-s**, WAM 92.718. **t**, WAM 97.936. **u**, *Metacrinus* sp. 1, WAM 84.597. Scale bars = 5 mm.

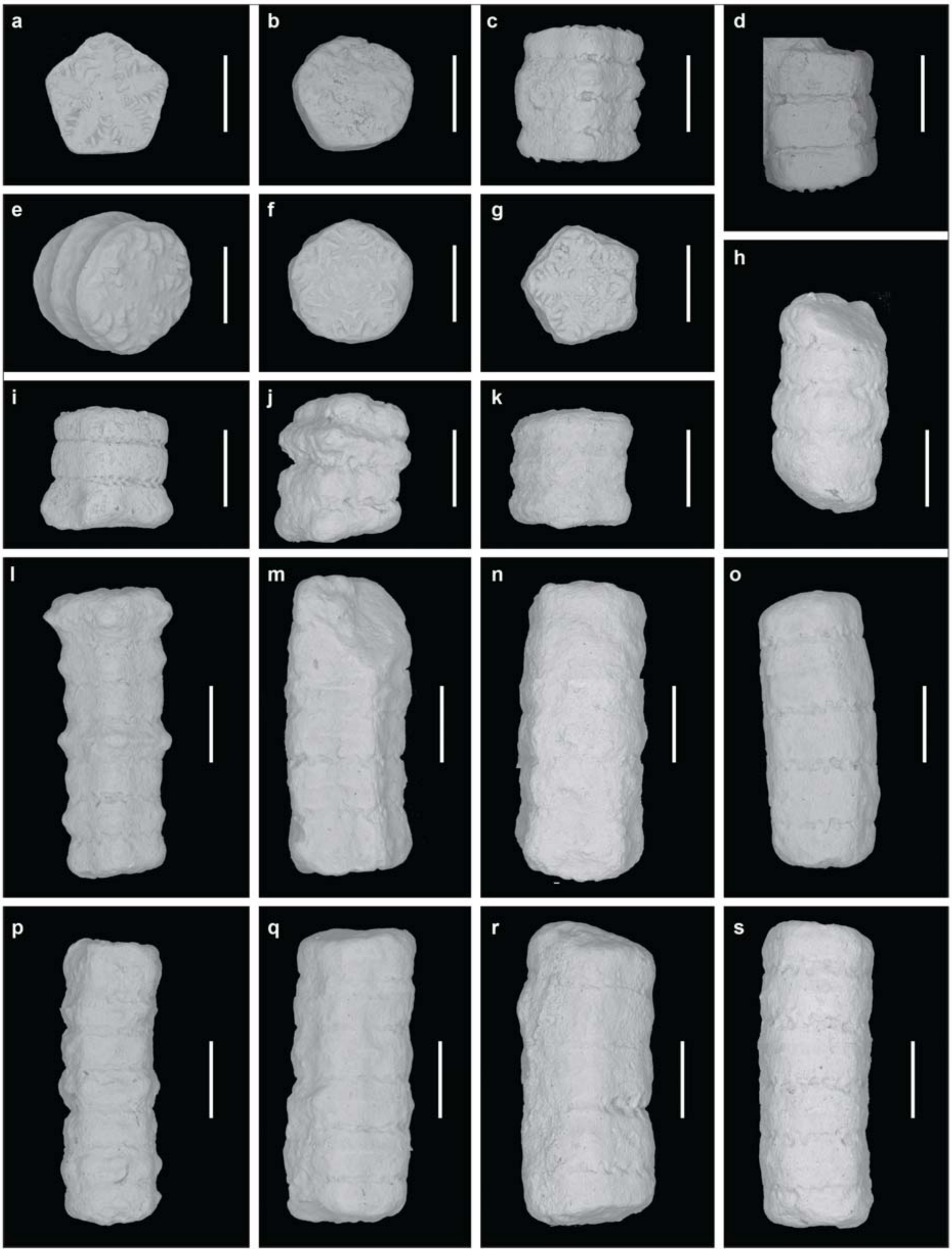

Supplementary Figure 2. New taxon from the Cardabia Formation (Wadera Calcarenite Member), Paleocene, Western Australia. a-s, *Isocrinus* sp. 4 WAM 17.842, WAM 17.861. lateral (a-b & e-g) or articular (c-d & h-o) surface views. Scale bars = 2 mm

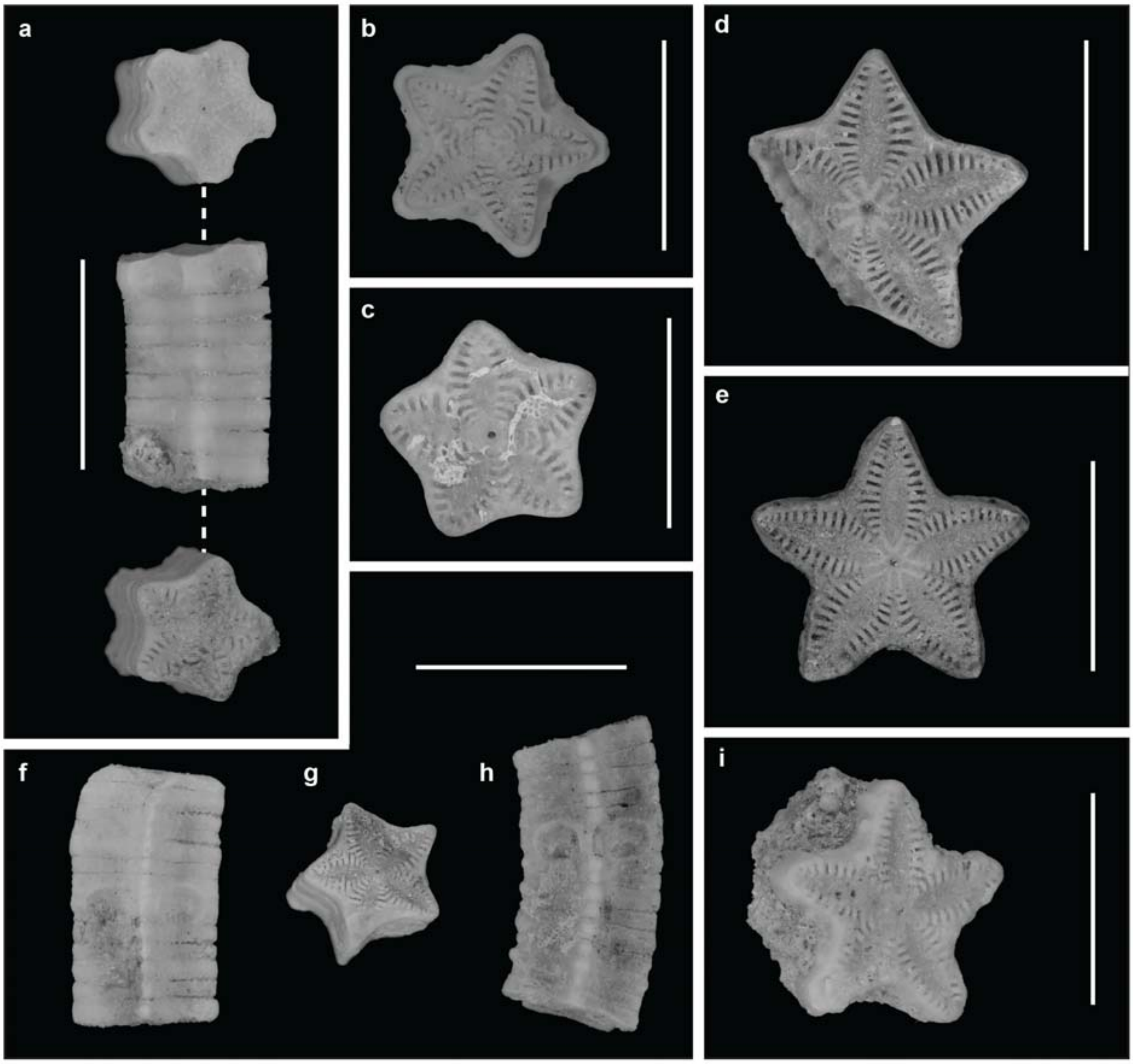

Supplementary Figure 3. New Metacrinids from the Nanarup Limestone and Wilson Bluff Limestone (Toolina Limestone) middle Eocene, Western Australia. a-c, & f-h, *Metacrinus* sp. 2. **a,** lateral or articular surface views WAM 87.223. **b & c,** articular surface views WAM 17.1938. **f-h,** lateral or articular surface views WAM 88.374a. **d, e & i,** articular surface views *Metacrinus* sp. 3. **d & e** WAM 18.1**.** **i**, WAM 17.1937. Scale bars = 10 mm.

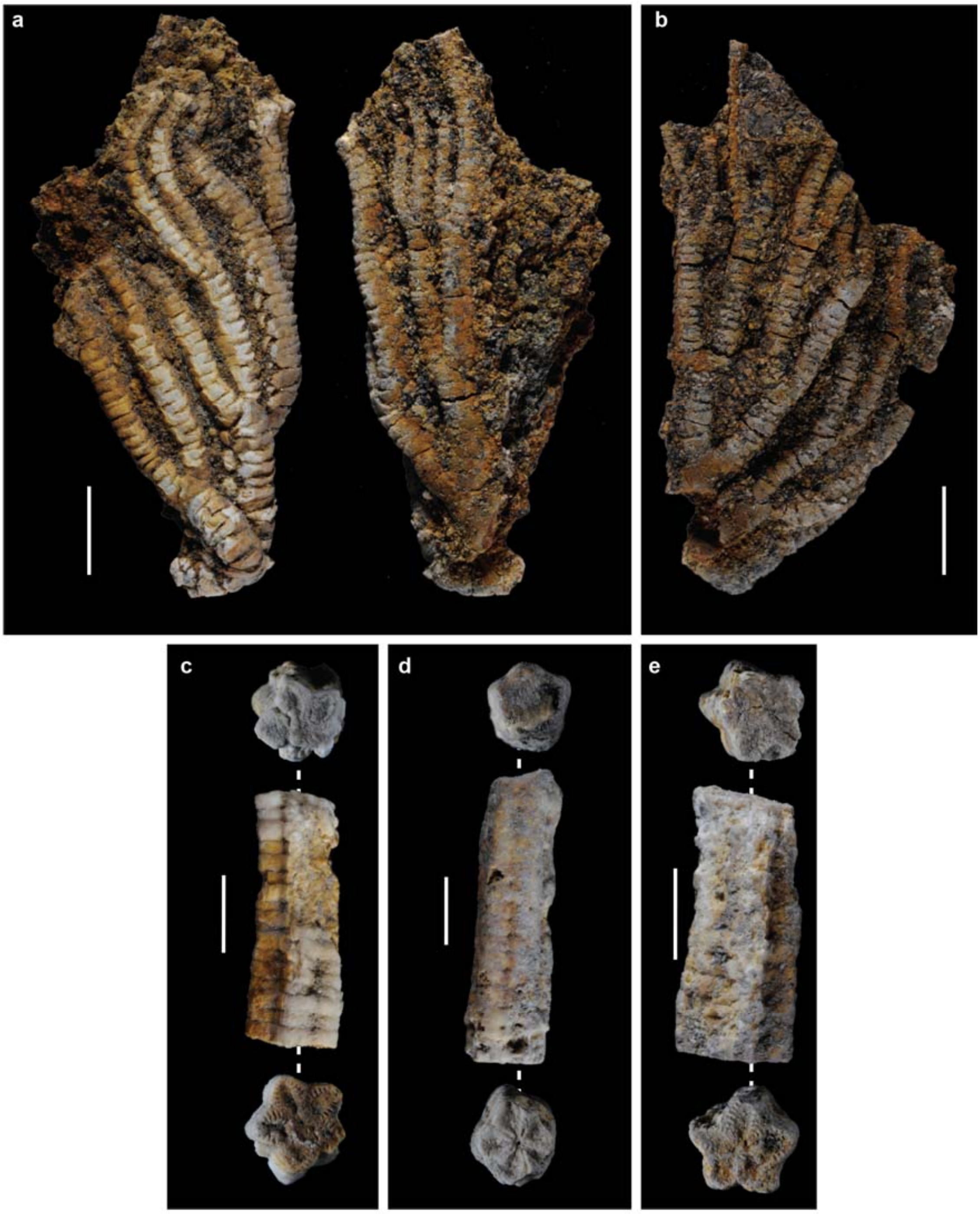

Supplementary Figure 4. Newly described Antarctic specimens. a,b, *Saracrinus* sp. from the Cross Valley Formation, Seymour Island, Antarctica, lateral surface views. Scale bars = 10 mm. **a,** specimen D.916.1. **b,** Specimen D.916.2. **c-e**, lateral or articular surface views *Metacrinus* sp. 4. from the Sobral Formation. Scale bars = 5 mm. **c**, D9.211.528. **d**, D9.211.530. **e**, D9.211.529.

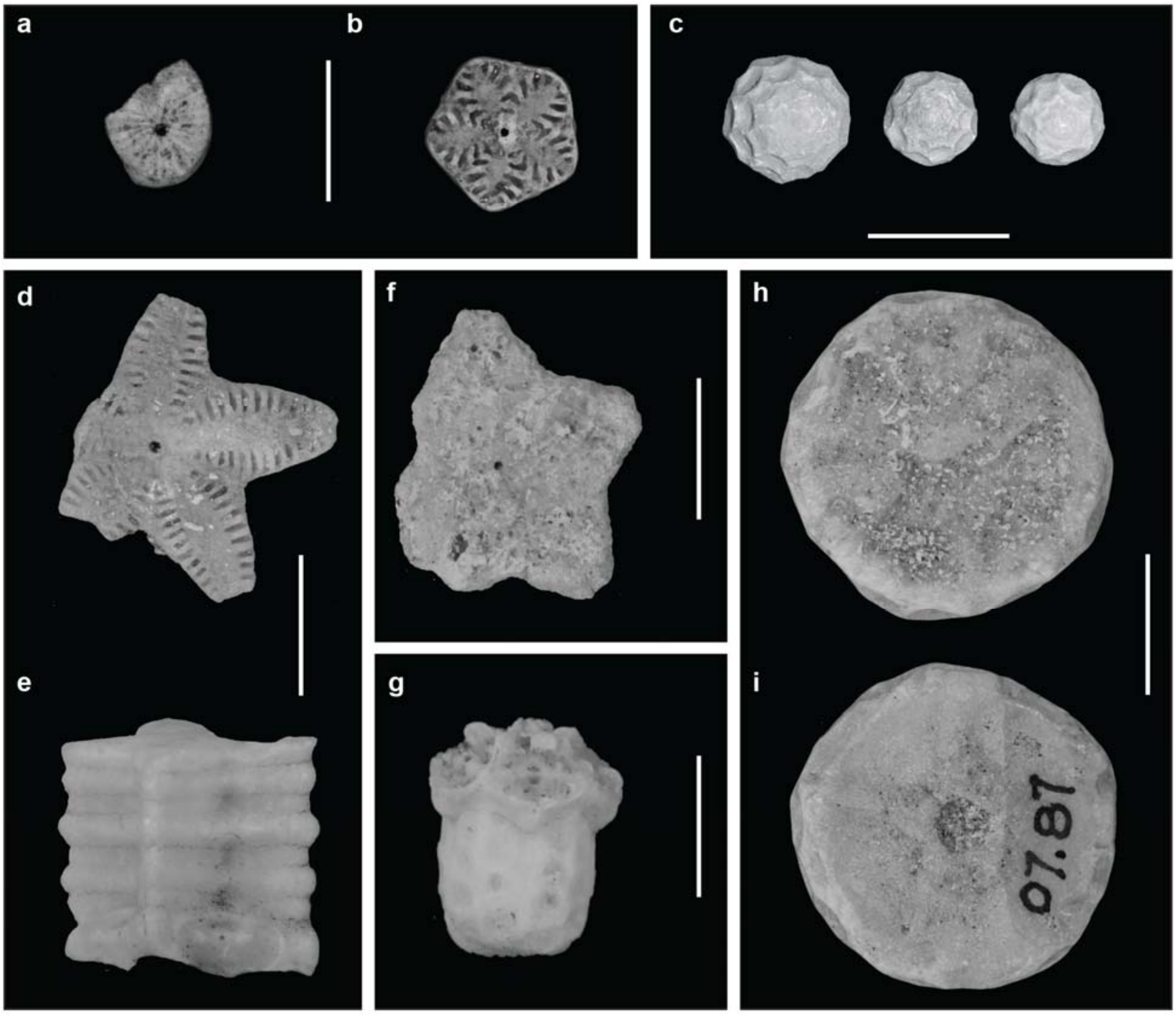

Supplementary Figure 5. New Australian taxa. a-b, *Isocrinus* sp. indet, articular surface view WAM 94.510, Browns Creek Clay, Eocene, Victoria, Australia. **d-e**, *Metacrinus* sp. 3, lateral or articular surface views WAM 06.238. **f**, *Metacrinus* sp. 2, articular surface view WAM 06.313 from the Tortachilla Limestone/Blanche Point Marl, Eocene, South Australia. **c, &** **g-i**, Comatulids from the Mannum Formation (Lower Beds), Miocene, Murray River, South Australia. **g**, *Loriolometra* sp. lateral surface view WAM 85.1252. **c**, *Notocrinus* sp., articular surface views NHM-UK EE1261-2. **h & i**, *Glenotremites* sp. articular surface views WAM 07.87. Scale bars = 5 mm.

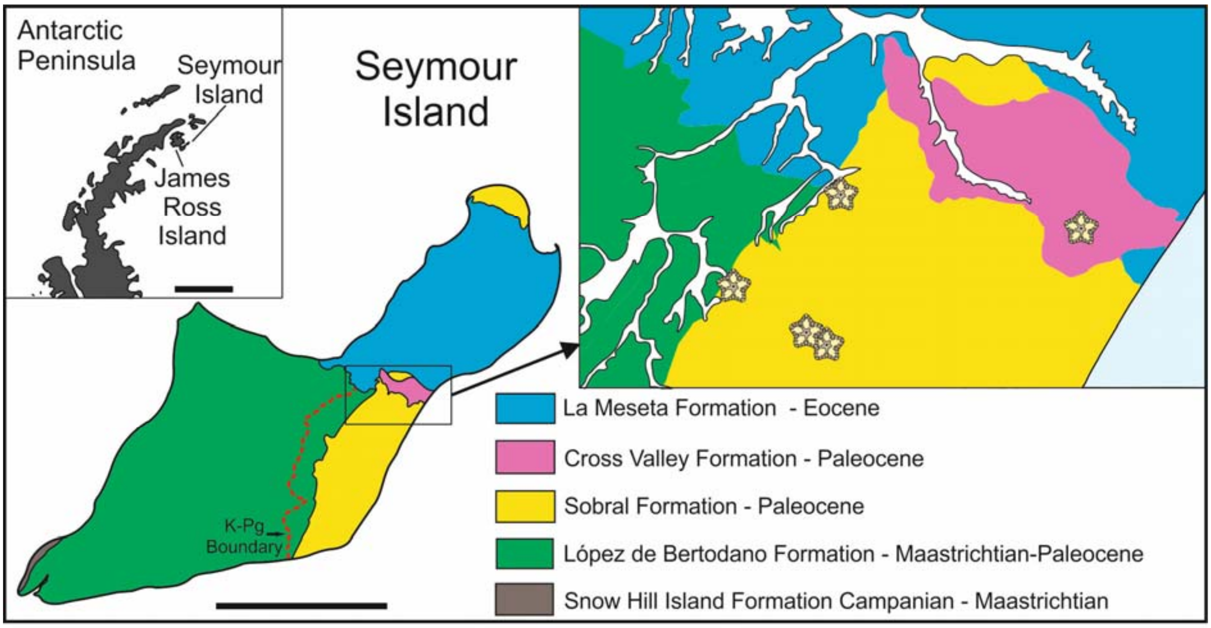

Supplementary Figure 6. Location and generalised stratigraphy of Seymour showing location of new crinoid specimens - *Metacrinus* sp. 4 from four localities in the Sobral Fm and *Saracrinus* sp. from one locality in the Cross Valley Fm. Map modified from Montes et al[1]. Antarctic Peninsula scale bar = 250 km, Seymour Island Scale bar = 5 km.

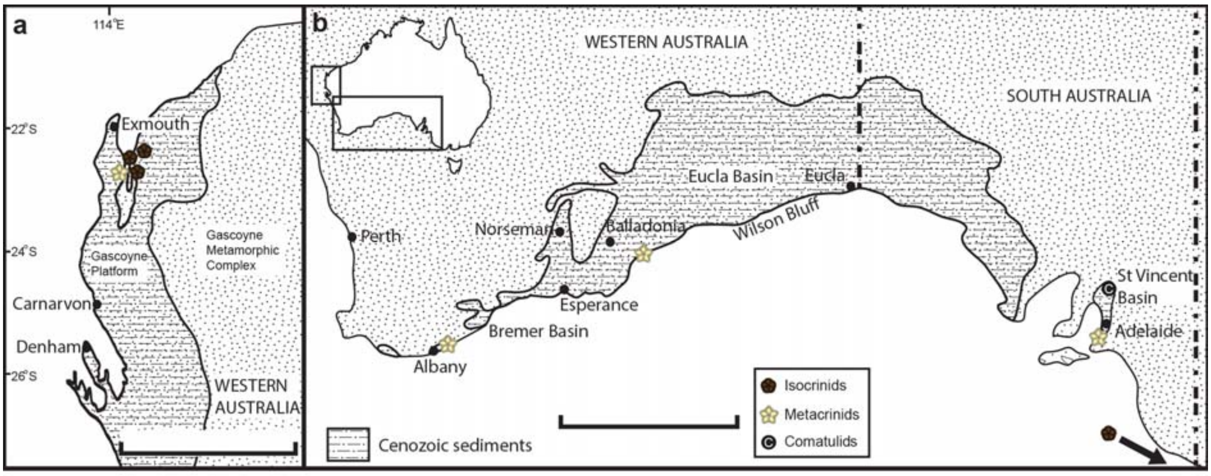

Supplementary Figure 7. Australian fossil localities. **a**, Location of the Australian Carnarvon Basin in Western Australia. Localities for *Isocrinus* sp. 1, *Isocrinus* sp. 2, *Isocrinus* sp. 3, *Isocrinus* sp. 4 and *Metacrinus* sp. 1 are shown. **b**, Australian Great Bight Basin which is subdivided into the Eucla and St Vincent basins. Scale bars = 500 km. Map outline modified from Seton et al[2].

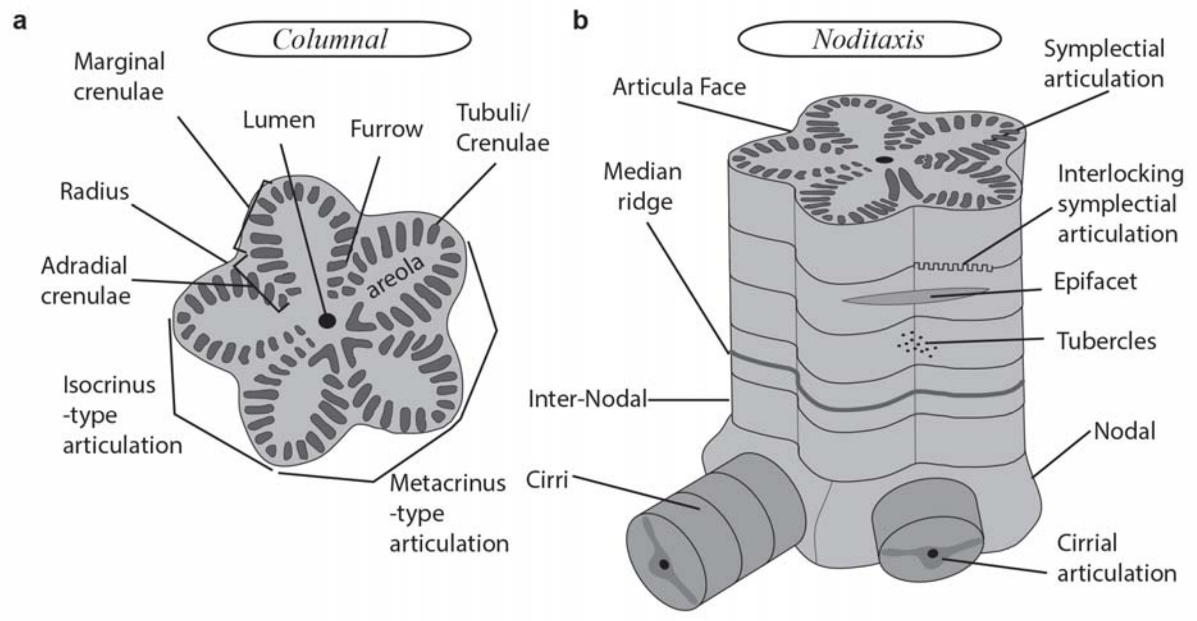

Supplementary Figure 8. Characters used in the systematics of crinoid columnals

(Redrawn from Hunter et al.[3]). **a.** Columnal features. **b.** Noditaxis features.

Supplementary Note 1: Systematic Palaeontology

Order ISOCRINIDA

Family ISOCRINIDAE Gislén 1924[4]

Subfamily ISOCRININAE Gislén 1924[4]

Isocrinus VON MEYER in AGASSIZ, 1836[5]

Diagnosis: Basals small, visible from outside, not forming contiguous circlet. Columnals low, except in distal-most part of column, cirrus sockets large, as high as nodals.

Remarks: Hess[6] separated this genus into two subgenera *I. (Isocrinus)* and *I. (Chladocrinus)* based on the structure of the articulation between secundibrachials 3 and 4; which is symmorphy between secundibrachials 3 and 4 for *I. (Isocrinus)*, and straight cryprosyzygy between secundibrachials 3 and 4 for *I. (Chladocrinus)*. With no cup material available for study, we assign our taxa based on the number of internodals.

I. (Isocrinus) Agassiz, 1836[5]

Description: Symmorphy cryprosyzygy between secundibrachials 3 and 4. Internodes short, with >10 columnals. Greater distance between nodals. Stem pentastellate-pentalobate to pentagonal. Nodals sub-pentagonal to pentalobate/pentastellate. Nodal-internodal junction has cryptosymplexy, all other articulations are symplectial. Facets of columnals (symplectial articulum) have elliptical petals, with gradual continuation of marginal and adradial crenulae reaching greatest length in radial marginal areas. Adradial crenulae of adjacent areolae

generally separated and at a slight angle to each other except near lumen. Cirral ossicles are circular.

Remarks: With no characters available for study in the arms, stem characters alone are used. Shorter internodal lengths of fewer than 10, typically >8 (mostly 7) columnals[7], except in proximal most part of the stem, suggest all these taxa belong to the *Isocrinus* subspecies, rather than the *Chladocrinus* subspecies that has intermodal lengths up to 20 columnals. Unlike *Metacrinus*, the size of the nodals and internodals is similar. Size of cirrus sockets can also be useful in distinguishing species.

I. (Isocrinus) sp. 1

Supplementary Figure 1 (a-e)

Description: Stem pentastellate to pentagonal. Internodes relatively short 9-10 columnals. Nodals are sub-pentagonal to pentastellate. Nodal-internodal junction has cryptosymplexy; all other articulations are symplectial. Nodals and internodals are medium to high. Latera are smooth and unornamented except for a small process on interradius. Cirrus sockets cover 2/3 of the nodal height and are deeply set pointing slightly downward distally. 3 Marginal crenulae, 3 fused adradial crenulae.

Occurrence: Cardabia Formation (Wadera Calcarenite Member), Paleocene, Western Australia. The taxon originally identified by Milner[8] as ?*Nielsenicrinus* sp. is now assigned to *I. (Isocrinus)* sp. 1 (see published occurrences below).

I. (Isocrinus) sp. 2

Supplementary Figure 1 (f-p)

Description: Stem pentastellate - pentagonal. Internodes short, 5-11 columnals. Nodals sub-pentagonal to pentastellate. Nodal-internodal junction has cryptosymplexy, all other articulations are symplectial. Nodals and internodals medium to high. Latera have a well-developed median ridge with an epifacet with process on inter radius and radius. 4 marginal crenulae and 3-4 adradial crenulae. Cirrus sockets are large and cover height of the nodal pointing slightly outward then downward distally.

Occurrence: Cardabia Formation (Wadera Calcarenite Member), Paleocene, Western Australia.

I. (Isocrinus) sp. 3

Supplementary Figure 1 (q-t)

Description: Stem sub-rounded to pentagonal. Internodes relatively short 7-8 columnals. Nodals sub-rounded-pentagonal. Nodal-internodal junction has cryptosymplexy, all other articulations are symplectial. Nodals and internodals medium height. Distal latera smooth and unornamented while the proximal latera have a distinct small process and ridge on interradius. Cirrus are very small sockets, covering less than 1/2 of nodal height pointing outward. 4 marginal crenulae and 3 adradial crenulae.

Occurrence: Cardabia Formation (Wadera Calcarenite Member). Paleocene, Western Australia.

I. (Isocrinus) sp. 4

Supplementary Figure 2 (a-s)

Description: Stem pentastellate to pentagonal. Internodes relatively short 8-10 columnals. Nodals pentagonal to pentastellate. Nodal-internodal junction has cryptosymplexy, all other articulations are symplectial. Nodals and internodals are medium height. Proximal latera are smooth and unornamented except for a very small process on interradius, while marginal and adradial crenulae not visible. Cirrus are very small sockets covering less than 1/2 of the nodal height pointing outward.

Occurrence: Cardabia Formation (Wadera Calcarenite Member). Paleocene, Western Australia.

I. (Isocrinus) sp. indet

Supplementary Figure 5 (a-b)

Description: Stem pentagonal. Internodals are medium to high. Latera are smooth and unornamented. 3 marginal crenulae and 3 adradial crenulae.

Occurrence: Browns Creek Clay, Eocene, Victoria, Australia

Remarks for I. (Isocrinus): These isocrinids are similar in having a small size, pentagonal to pentastellate stem and high columnals. However, there are significant differences in the shape of the latera with species 1 and 3 being much smoother than the highly ornamented species 2, and species 1 and 2 having cirral scars that are much bigger than species 3. The much smaller species 4 has smooth rounded to sub-pentagonal distal columnals with proximal columnals being far more ornamented with well-developed epifacet on the latera. There is insufficient taxonomic information on the 5th species to attempt further taxonomy.

Subfamily METACRININAE Klikushin, 1977[9]

Cryptosyzygy or synarthry between primibrachials 1 and 2, muscular articulation between secundibrachials 1 and 2. More than 2 primibrachials. Nodals with 5 cirrus sockets[9]. Roux[10] (p. 482) restricted the Metacrininae to only *Metacrinus* and *Saracrinus*, forms with more than 2 primibrachials. *Eometacrinus* Baumiller & Gaździcki (1996)[11] is similar to *Metacrinus* and *Saracrinus* in having 5 primibrachials and muscular articulation between secundibrachials 1 and 2, but it differs in the synarthrial articulation between primibrachials 1 and 2.

Metacrinus Carpenter, 1882[12]

Description: Cup low and wide. Basals generally large, forming contiguous basal circlet; lower edge with median projection covering interradial edge of uppermost columnals. Typically 7 primibrachials (range: 2 to 11). Arms divided at primibrachials 4 to 7 and further divided 2 more times. Cryptosyzygy between primibrachials 1 and 2; in species with 7 primibrachials also a cryptosyzygy between primibrachials 4 and 5 or primibrachials 5 and 6; in secondibrachitaxis cryptosyzygy between secundibrachials 2 and 3, Br 3 and 4, and more distal parts of the arms. Other brachial articulations muscular. No synarthry. All axillaries follow an oblique muscular articulation. First pinnule on primibrachial 2. Column pentagonal to rounded sub-pentagonal. Columnal facets similar to *Isocrinus*. Crenulae rather short. 5 to 13 internodals, fewer in proximal part of column. Nodals larger than inter nodals, with 5 rather large, elliptical to circular cirrus sockets facing outward to slightly upward. Cirri long.

Remarks: Although the columnal facets are similar to *Isocrinus, Metacrinus* has very low internodal heights and unlike *Isocrinus* the nodals are larger and higher with the cirrus sockets

which are deeply set and oval rather than circular. All these taxa have these characters as well as variation in the structure of the articular face.

Metacrinus sp. 1

Supplementary Figure 1 (u)

Description: Stem pentagonal to round. Internodes short 8 columnals. Nodals are pentagonal to rounded. Nodal-internodal junction has cryptosymplexy, all other articulations are symplectial. Nodals and internodals are medium low in height. 7-8 marginal crenulae and 4-5 adradial crenulae. Latera are smooth apart from a well-developed epifacet that continues around the inter radius forming a slight process. Cirrus cover the height of the nodal and are deeply set.

Occurrence: Cardabia Formation (Cashin Calcarenite Member), Eocene, Western Australia.

Metacrinus sp. 2

Supplementary Figure 3 (a-c, f-h), Supplementary Figure 5 (f)

Description: Stem pentagonal to round. Internodes short 7-9 columnals. Nodals are pentagonal to round. Nodal-internodal junction has cryptosymplexy, all other articulations are symplectial. Nodals and internodals are low in height. Latera are smooth apart from weakly developed epifacet that continues around the inter radius. The inter radius is better developed in the proximal part of the stem. On the articular face there are typically 5 adradial crenulae and 7 marginal crenulae. Nodal is up to twice the height of the internodal. Cirrus cover the height of the nodal and are deeply set. 10-11 marginal crenulae and 3 adradial crenulae.

Occurrence: Nanarup Limestone, middle Eocene, Western Australia; Tortachilla Limestone, Eocene and Blanche Point Marl, upper Eocene, South Australia.

Metacrinus sp. 3

Supplementary Figure 3 (d&e, i), Supplementary Figure 5 (d&e)

Description: Stem pentastellate to pentalobate. Internodes short 7 columnals. Nodals pentastellate to pentalobate. Nodal-internodal junction has cryptosymplexy, all other articulations symplectial. Nodals and internodals are low in height. Latera are smooth. On articular face adradial crenulae are fused to form ridges. There are 4 unfused adradial crenulae and 8 marginal crenulae. Nodal is up to twice the height of the internodal. Cirrus cover the height of the nodal and are deeply set. 10 marginal crenulae and 3 adradial crenulae.

Occurrence: Wilson Bluff, middle Eocene, Western Australia; Tortachilla Limestone, Eocene and Blanche Point Marl, upper Eocene, South Australia.

Remarks: The three species are clearly metacrinids, with nodals which are higher than the internodals. The individual species can be distinguished by the height of the internodals and the number of crenulae. Species 1 has a well-developed epifacet not observed in the other species. Species 2 most resembles living species with a large epifacet on the proximal part of the stem. Species 3 is distinct due to its larger size, stellate shape and smooth latera. Species 3 also has fused adradial crenulae which is not observed in species 1 or 2.

Metacrinus sp. 4

Supplementary Figure 4 (c-e)

Description: 12 columnal sections, cup and arms not preserved. Described from the columnals. Articular facet is weakly pentastellate to almost pentagonal to strongly pentalobate. 5-6 marginal crenulae and 3-4 adradial crenulae. Internodal height is long >20 with most preserved examples typically 14-16. Most internodals are smooth, however every 3rd-4th internodal is slightly larger and has an epifacet. This morphology results in an irregular size of the latera. Nodals slightly larger than internodals with cirri sockets pointing upwards.

Remarks: Rasmussen[13] was unsure assigning this form to the existing extant genus of *Metacrinus* due to the lack of cup material available. Although cup material is still not available for this study the new material has increased the amount of taxonomic information. 12 pluri columnals have been examined as well as the figured material of Rasmussen[13]. Due to the lack of cup or arm material, the diagnosis is restricted to the examination of columnal material. The main diagnostic features in this part of the crinoid are found in the shape of the articular facet and the number of internodals in each pluricolumnal. *M. seymouriensis* is similar to *Isocrinus (Isocrinus)* and *Isocrinus (Chladocrinus*) in the shape of the articular facet which is pentagonal to pentalobate. However, the nodal lengths in *Isocrinus (Isocrinus)* are generally fewer than 10 and typically 7. The Jurassic species of *Isocrinus (Chladocrinus)* tends to have longer nodal lengths of up to 20. These nodal-lengths are in-line with other members of the isocrinids such as *Tyrolecrinus* (3-7) *Balanocrinus* (7) *Raymondicrinus* (6-7) *Chariocrinus* (10), *Hispidocrinus* (15). In addition, there is no evidence that the columnals are either typically stellate although those proximal columnals close to the cup are likely to be so, or sub-rounded to rounded as in *Isselicrinus*, *Austinocrinus*, *Cenocrinus,* and *Doreckicrinus*. *M. seymouriensis* very much resembles the modern tropical and sub-tropical genus of *Endoxocrinus* in being pentagonal to pentalobate. However, the inter-nodal lengths are highly variable, 3-16, with up to 30 *Endoxocrinus (Diplocrinus)*, while *M. seymouriensis* is more consistent. Re-examination

of Recent representatives of the genus *Metacrinus* confirm Rasmussen's[13] diagnosis. The stem is pentagonal to pentalobate although the number of nodals is slightly longer than that typical of the genus (13) with the nodals being slightly larger than the inter nodals. There are significant differences in the shape of the latera between species of *Metacrinus*. *Metacrinus rotundus* has smooth latera while *M. seymouriensis,* and *Metacrinus wyvillei* have more irregular latera. Specimen 211.528 has well preserved crenulae.

Occurrence: Seymour Island, Antarctica, Sobral Formation. Specimens occurred at three stratigraphic heights, occurrences were dated at ~ 65.4 Ma; ~ 65.2 Ma and ~ 64.95 Ma (ages from Bowman et al. [14]).

Saracrinus A. H. Clark, (1923, p. 9)[15]

Diagnosis: Cup low and wide. Typically 4 primibrachials (3-6) and always more than 2. Arms divided at primibrachials 3-6 and further divided two or more times. Cryptosyzygy only between primibrachials 1 and 2. Muscular articulation between secundibrachials 1 and 2. Cryptosyzygy between secundibrachials 3 and 4; all other brachial articulations muscular. No evidence of synarthy. All axillaries follow an oblique muscular articulation. First pinnule on primibrachial. Column not preserved.

Remarks: Typically 4 primibrachials (range: 3 to 6), only primibrachials 1 and 2 united by cryptosyzygy. This genus is closely related to *Metacrinus* but is treated herein separately in accordance with the recent literature[16,17,18].

Saracrinus sp.

Supplementary Figure 4 (a-b)

Diagnosis: As for genus

Description: Dorsal cup low, wide. Suture between radial and first primibrachial gently concave distally. IBr2 presumably cryptosyzygial or synostosial, although suture has small ridge-like distal projection. Articular facet unknown. Other primibrachial articulations oblique, muscular, except for IBr4 which is flat cryptosyzygial or synostosial, usually with tightly fused appearance, IIBr, cryptosyzygial (3+4). Dorsal surface of brachials smooth. Distal brachials almost cylindrical in dorsal view. Isotomous branching up to quartibrachs; further division series unknown. Most specimens with only several uppermost columnals attached to base of calyx; columnals strongly tapering. A small specimen with crown 31 mm long has only the proximal stalk; another small specimen has at least 7 mm of stalk with many cirri attached.

Remarks: Although no stem ossicles have been discovered and the radial and basal ossicles have not been preserved, the two specimens have a well-preserved crown fragment which exhibits most of the arms apart from the distal end of the primibrachials. It is clear that they have more than 2 primibrachials (3-4) so that these can be assigned to the subfamily Metacrininae. In addition, there are enough characters on the well-preserved arms to assign this taxa to *Saracrinus*. In all the arm sections examined, there are a maximum of 4 primibrachials (IBr) while in *Metacrinus* there are typically 7 primibrachials. Roux[10] restricted this subfamily to the genera *Metacrinus* and *Saracrinus*, which are similar in terms of distal arm structure and only distinguished by the number of primibrachials (BrI), 4 in *Saracrinus* and typically more than 7 in *Metacrinus*. Hess[6] retained this distinction in the Treatise of Invertebrate Paleontology, namely that *Saracrinus* is closely related to *Metacrinus*. Therefore, this specimen can be assigned to *Saracrinus*. It is unlikely to be *Eometacrinus* as there is no evidence of a synarthrial articulation between primibrachials. The only living species of

Saracrinus is *S. nobilis,* from the western Pacific therefore this is the first confirmed description of *Saracrinus* from the fossil record. *Saracrinus motuketeensis* was described from the early Miocene of New Zealand[19], however, this record was only based on stem ossicles. The features which distinguish *Saracrinus* from *Metacrinus* are found on the arms and thus *Saracrinus motuketeensis* is regarded herein as *Metacrinus motuketeensis*.

Occurrence – Cross Valley Formation Seymour Island, Antarctica, (Unit 25 of Montes et al.[1] - 'dark shales and fine sandstones with fauna of siliciclastics oysters, molluscs and birds' deposited after underlying volcaniclastic lithologies). Paleocene – Thanetian.

Order COMATULIDA

Family COMATULIDAE J. Fleming 1828[20]

Subfamily COMASTERINAE A. H. Clark, 1908[21]

Synarthry or cryptosynarthry between primibrachials 1 and 2. Arms divided at primibrachial 2 and either secundibrachial 2 or 4; further brachitaxis of 2 or 4 ossicles. Almost all species have more than 20 arms.

Superfamily NOTOCRINOIDEA Mortensen, 1918[22]

Centrodorsal conical, truncated conical, or hemispherical to discoidal. Aboral area in adult specimens generally cirrus-free or with traces of obliterated cirrus sockets, commonly rugose. Most genera with 5 pits arranged radially around apex, forming dorsal star, in large specimens commonly in a depressed area. Some specimens also with shallow, indistinct, lanceolate interradial impressions around aboral pole. Centrodorsal cavity narrow to moderate, 20 to 30 percent of centrodorsal diameter, always surrounded by, or exceptionally fused with, 5 radial pits housing coelomic extensions outside chambered organ and nerve capsule of centrodorsal cavity. Cirrus sockets generally large, with more or less distinct articular tubercles, and in most fossil genera with marginal crenulae. Sockets in 10 columns on small or conical centrodorsals, increasing in number during growth and tending to form 20 columns (obliterated on aboral apex) on hemispherical to discoidal centrodorsals of larger specimens of *Glenotremites* and *Remesimetra*. Sockets small in *Semiometra*, without distinct sculpturing, and closely alternating, without forming columns on a very low discoidal centrodorsal. Rod-shaped basals commonly exposed interradially, with or without subradial cleft. Radials with low exposed surface or concealed; distal margin may be concave, reaching edge of centrodorsal only inter

radially. Radial articular facet generally rather low and wide, but in *Semiometra* high. Muscle fossae distinct, similar in size to interarticular ligament fossae or higher. Radial cavity narrow to wide and funnel shaped. Arms 10, divided at primibrachial 2. Synarthry between primibrachials 1 and 2 and secundibrachials 1 and 2. Syzygy between secundibrachials 3 and 4 and distally at variable intervals. Arms and pinnules aborally rounded, not carinate. Pinnules short. No distal comb or aboral crest on oral pinnules. Modern genera viviparous and with ambulacral covering plates moderately developed or absent. Mouth central.

Family NOTOCRINIDAE Mortensen, 1918[22]

Diagnosis: Moderate to very large Notocrinoidea with conical, truncated conical, or hemi spherical to discoidal centrodorsal, commonly wider and aborally more flattened or concave in large specimens. Aboral side of centrodorsal with a distinct dorsal star except in *Schlueterometra* and adult specimens of *Notocrinus*. Commonly with shallow interradial impressions. Adoral side of centrodorsal with distinct, commonly large, and deep radial pits. Cirrus sockets in *Notocrinus* and *Semiometra* without distinct sculpturing, in other genera with distinct articular tubercles and marginal crenulae. Sockets large and in 10 to 20 columns or small and closely alternating without forming columns. Subradial cleft present or absent. Radials with low exposed surface, sometimes visible only inter radially or concealed. Radial articular facet rather steep and flat or concave.

Notocrinus Mortensen, 1917, p. 206[23]

Diagnosis: after Mortensen[23] Centrodorsal conical or truncated conical. Dorsal star consisting of 5 radial pores surrounding central pore or pit present in juvenile specimens. Cirrus sockets

without distinct sculpturing, in 10 columns, or in large specimens crowded, tending to form 20 columns. Aboral apex cirrus-free, pointed or truncated, rugose. Adoral side of centrodorsal with 5 simple, deep radial pits. Cirri stout, rather long. Cirrals rather short and wide; distal cirrals laterally compressed. Rod-shaped basals stout, centrally united, exposed inter radially or concealed. No subradial cleft. Exposed surface of radial low to moderate. Radial articular facet rather steep and wide, not angularly bent or concave. Adoral muscle fossae rather wide, triangular, larger than interarticular ligament fossae; adoral edge wide, with a broad and shallow median embayment. Radial cavity moderate. Arms large. Distal syzygies at interval of 2 to 20.

Notocrinus sp.

Supplementary Figure 5 (c)

Diagnosis: A small *Notocrinus* with small or no free dorsal surface and radial edge projecting downward in mid radial area between the uppermost cirrus sockets.

Description: Centrodorsal conical to subconical: sides straight to gently convex. Centrodorsal maximum width about 5.5 mm at the level of radials, height about 6 mm. Cavity 1 mm in diameter. Sides of centrodorsal covered by large cirrus sockets; on the single, clearly exposed radial area of centrodorsal, sockets not arranged in distinct columns or rows, closely spaced. Sockets circular, smooth, no articular ridge, no crenellae, relatively shallow, with a small axial pore. Cirri not preserved; Basal surface is flat and relatively unornamented. Dorsal ligament fossa large with a distinct ligament pit. Central canal large and sub-circular in outline.

Remarks: Very similar to the species described by Baumiller & Gaździcki[11], with comparable shape and arrangement of large cirrus sockets, single radial area of centrodorsal, and irregular

arrangement of the sockets. However, after examining 15 specimens, the width and height is at least double the size.

Occurrence: Mannum Formation (Lower Beds), Miocene, Murray River, South Australia

Glenotremites sp.

Supplementary Figure 5 (h&i)

Diagnosis: Adapted from Rasmussen[24]; Centrodorsal is pentagonal to rounded and very low arched, almost flat, with sloping sides and a central depression with a radial dorsal star surrounded by cirrus sockets. They form 2 radiating rows in each of the five sides, those near the dorsal star are four large and deep concave sockets in each radial section with an elevated edge and a marginal rim of radiating crenellae.

Description: Centrodorsal hemispherical to discoidal: sides straight to gently convex. Centrodorsal maximum width about 13-14 mm at the level of radials, height about 8 mm. Cavity 2-3 mm in diameter. Sides of centrodorsal covered by large cirrus sockets (approx. 10-15); sockets not arranged in distinct columns or rows, and are slightly spaced. The exposed radial area of the aboral surface of the centrodorsal tends to be flattened unornamented (naked). Sockets circular, smooth, no articular ridge, some crenellae, shallow, with a small axial pore. Cirri not preserved; basals surface flat with distinct dorsal ligament fossa, large, and a distinct ligament pit. Central canal large and sub-circular in outline.

Remarks: Glenotremites tends to have a flat 'button shaped' centrodorsals. Unlike most *Glenotremites*, this species tends to be larger and has a flattened unornamented aboral surface.

Occurrence: Mannum Formation (Lower Beds), Miocene, Murray River, South Australia.

Loriolometra Gislén, 1924, p. 167[4]

Diagnosis: Centrodorsal large high, columnar or slightly conical with rounded aboral apex bearing an aboral pit or dorsal star. Faint interradial aboral impressions may be present. Cirrus sockets large, with a wide axial canal, lateral articular tubercles, and marginal crenulae. Sockets arranged in 10 distinct columns. Centrodorsal cavity approximately 20 percent of centrodorsal diameter. Radial pits simple, very wide and deep, exceeding depth of centrodorsal cavity, rod-shaped basals exposed interradially and separated around central canal. Radials forming complete contiguous exposed surface around centrodorsal. Deep subradial cleft. Radial articular facet steep, almost vertical with large and wide, triangular aboral muscle fossae. Radial cavity wide.

Remarks: Previously only know from the Late Cretaceous (Campanian) of Sweden and France, our assignment is based on the elongated conical shape and distinctive rugose aboral surface.

Loriolometra sp.

Supplementary Figure 5 (g)

Diagnosis: As for genus

Description: Centrodorsal long and elongate: sides straight. Centrodorsal small, centrodorsal maximum width about 4-5mm at the level of radials, height about 9 mm. Cavity 2-3 mm in diameter. Sides of centrodorsal covered by large cirrus sockets (approx. 10); sockets arranged in rows, and are slightly spaced. The exposed radial area of the aboral surface of the centrodorsal is slight flattened and rugose with large aboral pit. Sockets circular, smooth, no articular ridge, some crenellae, shallow, with a small axial pore. Cirri not preserved; basals surface flat, with distinct dorsal ligament fossa large and distinct ligament pit.

Remarks: This species is slightly smaller than those examined from the Cretaceous. However, it still has the distinctive conical shape with a rugose aboral surface typical of the genus, but the arrangement of the cirrus sockets is more regular than other members of the genus.

Occurrence: Mannum Formation (Lower Beds), Miocene, Murray River, South Australia.

Supplementary Note 2: Geological Settings

The subsequent section summarises the geological setting of the above described crinoids, with the current interpretation of the environment of deposition. Herein shallow water is defined as occurring on the inner shelf or shallower.

Antarctic Specimen Geological Settings

Unit: Sobral Formation

Location: Antarctic Peninsula, Seymour Island (Supplementary Figure 6).

Age: Paleocene

Taxon: *Metacrinus* sp. 4

Environment: *Shallow marine delta*

The contact at the base of the Sobral Formation has been dated at 65.5 Ma[25]. The **Sobral Formation** was deposited in the Paleocene, and shows an overall coarsening-upward, regressive trend reflecting eastward progradation of a marine delta[26,27] that is interpreted to have been wave-dominated but influenced by tidal processes in the shallow, proximal regions (Ineson pers comm). *Metacrinus* specimens were found in association with bivalves (*Leionucula*, *Cucullaea*, *Periploma*, *Pycnodonte*, *Lahillia*, *Neilo* and *Marwickia*/*Cyclorismina*), gastropods ('*Vanikoropsis'*, *Struthiochenopus*, *Euspira*?, *Heteroterma*, *Marshallaria*, *Miomelon*? and *Taioma*), cidaroid echinoid spines, solitary and branching corals, scaphopods and brachiopods (*Bouchardia*)[28] (Whittle pers comm). The

Sobral Fm has been interpreted as a shallow water unit, deposited by the filling of the James Ross Basin by progradation of a delta, with sediments originating from the Antarctic Peninsula[26]. This occurred in a wave dominated tide influenced system (Ineson pers comm).

Unit: Cross Valley Formation

Location: Antarctic Peninsula, Seymour Island (Supplementary Figure 6).

Age: Paleocene

Taxon: *Saracrinus* sp.

Environment: *Very shallow marine*

The Paleocene **Cross Valley Formation**, is a valley infill deposit, which cuts into the lower Paleocene Sobral Fm and older beds[29,30]. A Late Paleocene age for the upper Cross Valley Formation was suggested based on dinoflagellates and pollen[31,32]. Three "allomembers" are distinguished[1,27]; the crinoid specimens were collected from the base of allomember C. Allomember A is dominated by volcanic clasts, conglomerates and glauconitic sandstones. This lower part of the formation has been interpreted as a lahar deposit[33]. Allomember B consists of sandstones with charred wood and leaf imprints, interpreted as deposited in an estuary with tidal influence[1]. Allomember C consists of sandstones and shales, some of which contain a fauna of oysters, fish teeth, sharks, gastropods and also a penguin that was described by Tambussi et al.[29]. Tambussi et al.[29] mention crinoids from this allomember but, until now, no specimens have as yet been described from this unit. Other beds contain plant imprints[1,27]. Deposition is thought to have occurred in a very shallow marine environment with a potential decrease in sea level, followed by a transgression[27].

Australian Specimen Geological Settings

Unit: Cardabia Formation **-** Wadera Member

Location: Western Australia, Northern Carnarvon Basin, Giralia Anticline (Exmouth Gulf) (Supplementary Figure 7a).

Age: Paleocene

Taxa: *Isocrinus* sp. 1*, Isocrinus* sp. 2*, Isocrinus* sp. 3*, Isocrinus* sp. 4.

Environment: *Shallow marine (within the photic zone)*

The **Cardabia Formation,** originally known as the "Cardabia Group", was reduced to formation status by Hocking et al.[34], and the constituent "formations" to member status. Named members within the formation (where they can be differentiated) are, in ascending order: the Boongerooda Greensand, Wadera, Pirie, Cashin, and Jubilee Members. The Boongerooda Greensand, Wadera and lower parts of the Cashin members are Paleocene, while the upper part of the Cashin member and Jubilee Members are Eocene. In the Giralia Anticline, the lithology of the Cardabia Formation, following Hocking et al.[34], consists of a basal greensand (the Boongerooda Greensand Member), overlain by calcarenite and calcisiltite. The sequence is interpreted as an unconformity bounded, single depositional episode, in the onshore area. The calcareous bryozoal calcarenite indicates close proximity to the shoreline. The Formation is richly fossiliferous and has yielded faunas consisting of planktic and benthic foraminifera, bryozoans, ostracods, echinoids, brachiopods, bivalves, gastropods, nautiloids, crustaceans and crinoids. The basal member of the Cardabia Formation (the Boongerooda Greensand Member) is a condensed sequence, which formed in quiet, uniform, shelf conditions. The presence of glauconite and phosphate make a water depth of less than 30 m unlikely[35,36]. The **Wadera**

Member is a hard, well-bedded, yellowish, fine- to medium-grained, glauconitic, calcarenitic grainstone to packstone, interbedded with soft marly limestone. Bryozoans are abundant towards the top of the Wadera Member. Distinctive smoothly curving burrows occur throughout the unit, small corals suggest a shallow marine environment within photic zone.

Unit: Cardabia Formation **-** Cashin Member

Location: Western Australia, Northern Carnarvon Basin, Giralia Anticline (Exmouth Gulf) (Supplementary Figure 7a).

Age: Eocene

Taxa: *Metacrinus* sp. 1.

Environment: *Shallow marine open shelf*

The **Cashin Member** of the Cardabia Formation consists of calcarenitic packstone to grainstone, with thin to medium scale, nodular to regular bedding. The Cashin Member probably formed in an open-shelf setting, as migrating shoals of calcarenite, in a generally calcilutitic- calcisiltitic environment.

Unit: Werillup Formation, Nanarup Limestone Member

Location: Western Australia, Western Eucla Basin, Part of Great Bight Basin (Supplementary Figure 7b).

Age: lower to middle Eocene

Taxa: *Metacrinus* sp. 2

Environment: *Shallow marine environment during a lull in detrital deposition*

The **Nanarup Limestone Member** is a distinct unit of the Werillup Formation (the lower unit of the Plantaganet Group). It comprises brown and white friable bryozoan limestone. The limestone has a rich fauna of foraminifers, echinoids, bryozoans, brachiopods, and molluscs. It is considered to correlate with the Wilson Bluff, Paling and Norseman Formations in other parts of the Eucla Basin[37], and with the Tortachilla Limestone in the St Vincent Basin in South Australia. These carbonate units were all deposited during planktonic foraminiferal zones P14–15, and are thus Bartonian (late middle Eocene) in age[38]. They were deposited in the Tortachilla transgressive event[37] and their occurrence marks the initiation of carbonate deposition off the southern margin of Australia, which correlates with the acceleration in the divergence of Australia from Antarctica. The fauna of the Nanarup Limestone suggests that it was deposited in a shallow marine environment during a lull in detrital deposition, enabling a shelly carbonate limestone to form[39].

Unit: Wilson Bluff Limestone (also known as Toolina Limestone).

Location: Western Australia – Eastern Eucla Basin, part of the Great Bight Basin (Supplementary Figure 7b).

Age: middle Eocene

Taxa: *Metacrinus* sp. 3

Environment: *Shallow Marine Shelf*

The Eucla Basin is the largest onshore example of Cenozoic marine sediments in the world. The basin extends 2000 km from east to west and, including offshore extensions, 500 km from north to south. The **Wilson Bluff Limestone** was named by Singleton[40] for the soft fine-grained chalky limestone rich in bryozoans and scattered echinoid tests and spines[41]. Locally

brachiopods, bivalves, and sponge spicules are abundant. The unit is exposed in the lower part of the coastal cliffs of the Nullarbor Plain, with the type section at the South Australian/Western Australian border. Planktic foraminifera suggest a middle Eocene age and an outer shelf deposition (70–>100 m) for this unit[41]. However, the presence of grainstones and local patchy development of typical shallow water invertebrate faunal elements, suggest a locally shallower depositional environment for this facies[37].

Unit: Tortachilla Limestone

Location: South Australia – St Vincent Basin (Supplementary Figure 7b).

Age: middle Eocene

Taxa: *Metacrinus* sp. 2*, Metacrinus* sp. 3.

Environment: *Shallow marine with hardgrounds*

The **Tortachilla Limestone** and the **Blanche Point Formation** are part of Eocene-Oligocene outcrops along Maslin and Aldinga Bays, near Port Willunga in South Australia. The Tortachilla Limestone is a yellow-brown, green and grey bioclastic autochthonous limestone, which grades from the base upwards from bryozoan sands rich in limonite grains into a hard, richly fossiliferous bryozoan limestone, which becomes partly glauconitic towards the top[42]. The colour also grades from the base to the top from brownish to pinkish-white. The top of the formation is a recrystallized limestone with quartz grains and limonite pellets[43]. There is a deeply corroded hardground into which softer glauconitic clays of the overlying Tuketja Member have percolated down and stained the limestone[43-45]. The Tortachilla Limestone is middle Eocene (Bartonian), planktic foraminiferal zones 14-15, in age[43,46].

Unit: Blanche Point Formation

Location: South Australia – St Vincent Basin (Supplementary Figure 7b).

Age: late Eocene

Taxa: *Metacrinus* sp. 3

Environment: *Shallow marine with hardgrounds*

The **Blanche Point Formation** was recently approximately correlated with planktic Zone P 16-17[47]. It disconformably overlies the Tortachilla Limestone, the basal **Tuketja Member**, where the crinoid specimens were collected, is a clayey limestone with hardened calcareous intervals[44]. Glauconite is abundant, giving the outcrop a greenish speckled appearance. A late Eocene age[38] is indicated by the foraminifera *Hantkenina primitiva* Cushman & Jarvis[48,49]. The dominance of the cytherellids in the Tuketja Member is mainly brought about by a single species, namely *C. gullrockensis* McKenzie, Reyment & Reyment[50]. This species is of the consueta group, and tends to typify outer shelf or deep-water assemblages (cf.[50-52]); however the presence of hardgrounds and a co-occurring shelly fauna are indicative of a shallow water setting[43].

Unit: Mannum Formation

Location: South Australia – Murray Basin (Supplementary Figure 7b).

Age: Miocene

Taxa: *Glenotremites sp., Notocrinus sp., Loriolometra sp.*

Environment: Shallow subtidal (euphotic zone), neritic depositional conditions

The **Mannum Formation** forms the middle and lower part of the Murray Supergroup, and consists of lower and upper members separated by the Swan Reach Dolomite Member. The formation consists of a finely bioclastic to marly, to medium- to coarse-grained, quartzose (<10%), bioclastic calcarenites, which occur as (i) pervasively bioturbated, bedded on a 10-90 cm scale delineated by burrowed omission surfaces, or (ii) low-angle trough cross-stratified, or hummocky cross-stratified beds (40-150 cm thick) with minimal bioturbation[46,53]. Mannum Formation limestones are typically well-cemented and dominated by echinoids and comatulid crinoids, except the upper beds which possess abundant cellarid bryozoans and crustose coralline algae[54]. Of the three members, the lower and upper members remain informal, based on their relatively poorly defined distribution and extent in the subsurface. These members are defined primarily on their dominant palaeontological attributes for ease of identification in outcrop[53]. Limestones of the Mannum Formation represent. Echinodermal calcarenite of the lower member was deposited under relatively high-energy conditions. While elsewhere, the limestones are heavily bioturbated suggesting a lower energy environment. The bryozoan-foraminiferal grainstone of the upper member represents deposition under open-marine, warm-temperate conditions[55]. Dominant macrofauna include the echinoids *Lovenia forbesi* and *Fibularia gregata*, bryozoans (*Sphaeropora* sp., *Porina* sp.), and the worm tube *Ditrupa*. Crinoid-rich lithologies were transgressive over granite. The lower boundary of the formation is typically conformable, grading from calcareous clay/marl and fine quartz sandstone of the underlying Ettrick Formation. The Upper Mannum Formation is dominantly calcarenitic, bedded on a 20-90 cm scale, possessing limonite-stained bioclastic grains and subordinate fine quartz sand. The upper formation limestone is also dominated by echinoids, but with no crinoids and far richer in bryozoans terebratulid brachiopods, coralline algae, and numerous species of bivalves and gastropods[46,53].

Unit: Browns Creek Clay

Location: South Australia – Victoria, Otway Basin (Supplementary Figure 7b).

Age: Eocene

Taxa: *Isocrinus* sp. indeterminate.

Environment: Neritic environment on the continental shelf

The **Browns Creek Clay** crops out in the coastal region of Victoria west of Cape Otway[55]. The Johanna River Sands, underlying the Browns Creek Clay lack calcareous microplanktic remains, but the contact between these two formations has been correlated with the sequence boundary between the third-order cycles 4.1 and 4.2 of Haq et al.[56] see Shafik[57]. The base of the Browns Creek Clay coincides with the first up-section appearance of calcareous nannofossils and planktonic foraminifera[57]. The biota appears immediately below a thin discontinuous calcarenite band in a small gully immediately west of Browns Creek (the 'first gully section' of Tickell et al. [58,59]). The basal ~8 m of the Browns Creek Clay at Browns Creek, the so-called Turritella clays, are predominantly dark grey clays, with common *Turritella* spp., *Spirocolpus aldingae* and the bivalve *Limopsis chapmani*. This is overlain by a prominent, 1–2 m thick bed of glauconitic sand, the *Notostrea* greensand that separates the Turritella clays from an overlying succession of marls and light grey clays[60]. The calcareous microplankton (nannofossil and foraminiferal) biostratigraphy and age of the lower part of the Browns Creek Clay are discussed in detail by Shafik[57]. Assemblages from the base of the formation include two key species, the nannofossil *Isthmolithus recurvus* and the foraminifera *Acarinina collactea*, in association with several important species, namely *Neococcolithes dubius*, *Chiasmolithus oamaruensis*, *Cyclicargolithus reticulatus*, *Discoaster saipanensis, Globigerinatheka* index, *Tenuitella aculeata*, *T. gemma* and *T. insolita*. The presence of *I. recurvus*, *C. reticulatus* and *D. saipanensis* suggests a late Eocene age, whereas the

foraminifera *Acarinina collactea* suggests a late middle Eocene age[60]. The older age is somewhat negated by the presence of the foraminifera *T. gemma* and *T. insolita*[57].

Previously Published Occurrences

New Zealand

Unit: Mantunau Group; Curiosity Shop Sandstone

Location: New Zealand, South Island, South Canterbury region, Rakaia Valley.

Age: lower Miocene (Otaian-Altonian)

Taxa: Hutton[61] described *Pentacrinus stellatus* from the Curiosity Shop Sandstone Oamaru Formation (Trelissiek Group). This has since been assigned to the undifferentiated Mantunau Group[62] found in in faulted outliers in the Canterbury Front. However this taxa is most likely to be an undescribed Isocrinidae gen. and sp. (Aaron Hunter pers comm).

Environment: Shallow marine sedimentation[63]

Unit: Mantunau Group; Waikari and Mount Brown Formation

Location: New Zealand, South Island, North Canterbury region, 'Pakau'.

Age: lower Miocene (Otaian-Altonian)

Taxa: Hutton[61] described *Pentacrinus rotates* from the Oamaru Formation (Ototara Group). This has since been assigned to the undifferentiated Mantunau Group[64] found in in faulted outliers in the Canterbury Front. This taxa is most likely to be an undescribed Isocrinidae gen. and sp. (Aaron Hunter pers comm).

Environment: Shallow marine sedimentation[63]

Unit: Waitemata Group; Cape Rodney Formation

Location: New Zealand, Auckland, Motuketekete Island

Age: lower Miocene (Otaian-Altonian)

Taxon*:* *Metacrinus motuketeketeensis* (Eagle 2004)[19] – Originally described as *Saracrinus*, this species is considered herein as *Metacrinus* – see Systematic Palaeontology section.

Environment: Inner shelf depths, associated with a diverse community of shallow- living, warm-water, marine invertebrates[20].

Unit: Otekaike Limestone Fm; Meyers Pass Limestone Member

Location: New Zealand, South Island, South Canterbury Region, Pentland Hills and Hurstlea.

Age: upper Oligocene (Duntroonian-Waitakian)

Taxon: Isocrinidae gen. and sp. not determined[65]

Environment: Shoal platform – Shallow Marine[65]

Unit: Torehina Fm

Location: New Zealand, North Island

Age: lower Oligocene (Whaingaroan)

Taxon: *Nielsenicrinus waiteteensis* Eagle 1993[66] (Family Isocrinidae).

Environment: Inner shelf marine palaeoenvironment, no deeper than 50 m[67].

Unit: Ototara Limestone

Location: New Zealand, South Island, Otago region,

Age: upper Eocene to lower Oligocene (Runangan-Whaingaroan)[67,68].

Taxa: 'Crinoid'[69], 'crinoid stem plates'[67]. Therefore likely to be Order Isocrinida.

Environment: Shallow warm water[67] submarine platforms in shallow marine shelf setting[70].

Unit: Island Sandstone

Location: New Zealand, South Island, North Westland,

Age: upper Eocene (Kaiatan-Runangan)

Taxa: Isocrinidae gen. and sp. not determined – 'part of an articulated crinoid with several cirri still attached'[71]

Environment: Shallow water, inner- or middle-shelf environment[71].

Unit: Kekerione Group; Red Bluff Tuff

Location: New Zealand, Chatham Islands

Age: upper Paleocene (Teurian) [63,72,73]

Taxa: *Isocrinus* cf *I. stellatus* (Hutton, 1873)[61,74]. Stilwell et al.[75] state that this fossil was discovered from an unknown locality from the Chatham Islands, however, there are many preserved echinoderm remains from the interbedded limestones within the Red Bluff Tuff[72]. Eagle[76] stated that this specimen was from Red Bluff Tuff at Waikari.

Environment: Shallow marine deposition in an embayment[72,73]

Unit: Onekakara Group; Kauru Formation

Location: New Zealand, South Island, North Otago region.

Age: middle Paleocene (Teurian)

Taxa: *Metacrinus* sp. was discovered in the Kauru Formation[75] which most likely part of the undifferentiated marine sediments of the Onekakara group of the north Otago region[77].

Environment: Shallow water[75]

South America

Unit: Leticia Formation

Location: South America, Tierra del Fuego, Patagonia

Age: upper–middle Eocene[78]

Taxa: *Isselicrinus* sp. [78]

Environment: Shallow marine[78]

Unit: Salamanca Formation

Location: South America, Patagonia

Age: lower Paleocene **(**Danian) [78]

Taxa: Unidentified[78], Metacrinidae – Aaron Hunter (pers comm)

Environment: Shallow marine[79]

Australia

Unit: Cardabia Formation (described as Cardabia Calcarenite)

Location: Carnarvon Basin, Western Australia

Age: Paleocene (mid Thanetian)[8]

Taxa: *?Nielsenicrinus* sp. (See *I. (Isocrinus)* sp. 1)[8]

Environment: The crinoid lived in an open shelf sea at 50 m to 100 m depth[8]

Antarctica

Unit: La Meseta Formation

Location: Seymour Island, Antarctica

Age: Eocene

Taxa: *Metacrinus fossilis*[16]

Eometacrinus australis[11]

Order Comatulida - *Notocrinus rasmusseeni*[16]

Order Comatulida - *Notocrinus seymourensis*[10]

Environment: Very shallow marine deltaic to estuarine environment[26]

Unit: Sobral Formation

Location: Seymour Island, Antarctica

Age: Paleocene

Taxa: *Isselicrinus antarcticus*[80]

Environment: Shallow marine (see description above).

Note: Rasmussen[13] described *Isselicrinus antarcticus* and *Metacrinus* (?) *seymouriensis* from the Sobral Fm and Cross Valley Fm, but noted these units as Maastrichtian. These units are both Paleocene and Maastrichtian deposits are also found on the island, so the exact locality for these specimens is uncertain.

Supplementary References

1. Montes, M., Nozal, F., Santillana, S., Marenssi, S. A. & Olivero, E. B. Mapa Geológico de la isla Marambio (Seymour) Escala 1: 20.000. *Argentino y Instituto Geológico y Minero de España, Instituto Antártico* (2010).
2. Seton, M., et al. Global continental and ocean basin reconstructions since 200 Ma. *Earth-Science Reviews* **113**, 212-270 (2012).
3. Hunter, A. W., Barras, C. G. & Thuy, B. Online field-guide to fossils: British Middle Jurassic echinoderms. *Proc Geol Assoc* **122**, 501–503 (2011).
4. Gislén, T. Echinoderm Studies: Academical Dissertation. *Zoologiska Bidrag fran Uppsala* **9**, 1–330 (1924).
5. Agassiz, J. L. R. Prodrome d'une monographie des Radiaires ou Echinodermes. *Mémoires de la Société d'histoire naturelle de Neuchâtel* 1 for 1835:168–199 (1836).
6. Hess, H., Messing, C. G. & Ausich, W. I. Revised, Crinoidea (3). *Treatise on Invertebrate Paleontology, Part T, Echinodermata 2*, ed Seldon, P. A. (The University of Kansas Paleontological Institute, Lawrence, Kansas), pp 1–261 (2011).
7. Oji, T. Early Cretaceous *Isocrinus* from northeast Japan. *Palaeontology* **28**, 661–674 (1985).
8. Milner, G. J. The first record of an isocrinid crinoid from the Tertiary of Australia. *Records of the Western Australian Museum* **14**, 385–389 (1989).
9. Klikushin, V. G. Sea lilies of the genus Isselicrinus. *Paleontological Journal* **11**, 87-95 (1977).
10. Roux, M. Echinodermes: Crinoïdes Isocrinidae. *Résultats des campagnes Musorstom I, Philippines (18-28 Mars 1976). Mémoires ORSTOM* **91**, 477–543 (1981).

11. Baumiller, T. K. & Gazdzicki, A. New crinoids from the Eocene La Meseta formation of Seymour island, Antarctic peninsula. *Acta Palaeontol Pol* **55**, 101–116 (1996).

12. Carpenter, P. H. Reports on the results of dredging under the supervision of Alexander Agassiz in the Gulf of Mexico (1877-78), in the Caribbean Sea (1878-79), and along the Atlantic Coast of the United States (1880) by the U.S. Coast Survey steamer "Blake". Lieut.-Commander C. D. Sigsbee, U.S.N., and Commander J. R. Bartlett, U.S.N., commanding. XVIII: The stalked Crinoids of the Caribbean Sea. *Bulletin of the Museum of Comparative Zoology Harvard* **10**, 165–181 (1882).

13. Rasmussen, H. W. Crinoideos del Cretacico y del Tertiario Inferior de la Isla Vicecomodoro Marambio (Seymour Island), Antartida. *Contribución del Instituto Antártico Argentino* **4**, 79–97 (1979).

14. Bowman, V. et al. The Paleocene of Antarctica: Dinoflagellate cyst biostratigraphy, chronostratigraphy and implications for the palaeo-Pacific margin of Gondwana. *Gondwana Res* **38**, 132–148 (2016).

15. Clark, A. H. A revision of the recent representatives of the crinoid family Pentacrinidœ, with the diagnoses of two new genera. *J Wash Acad Sci* **13**, 8–12 (1923).

16. Meyer, D. L. & Oji, T. Eocene crinoids from Seymour Island, Antarctic Peninsula: paleobiogeographic and paleoecologic implications. *J Paleontol* **67**, 250–257 (1993).

17. Améziane, N. & Roux, M. Biodiversity and historical biogeography of stalked crinoids (Echinodermata) in the deep sea. *Biodivers Conserv* **6,** 1557–1570 (1997).

18. Roux, M., Messing, C. G. & Améziane, N. Artificial keys to the genera of living stalked crinoids (Echinodermata). *Bulletin of Marine Science* **70**,799–830 (2002).

19. Eagle, M. K. *Saracrinus* (Crinoidea: Metacrininae) from the Early Miocene of Motuketekete Island, Hauraki Gulf, Auckland, New Zealand. *Records of the Auckland Museum* **41**, 5–12 (2004).

20. Fleming, J. *A History of British Animals, Exhibiting the Descriptive Characters and Systematical Arrangement of the Genera and Species of Quadrupeds, Birds, Reptiles, Fishes, Mollusca, and Radiata of the United Kingdom; Including the Indigenous, Extirpated, and Extinct Kinds, Together with Periodical and Occasional Visitants.* Bell & Bradfute, Edinburgh. 565 pp. (1828).

21. Clark, A. H. New genera of unstalked crinoids from the Philippine Islands. *U.S. National Museum Proceedings* **41**,171–173 (1908).

22. Mortensen, T. The" Crinoidea" of the Swedish Antarctic Expedition. *Wissenschaftliche Ergebnisse der Schwedischen Südpolar: Expedition 1901-1903* **4**,1–23 (1918).

23. Mortensen, T. *Notocrinus virilis* ng, n. sp. a new viviparous crinoid from the Antarctic Sea. *Videnskabelige Meddelelser Dansk Naturhistorisk Forening i København* **68**, 205–208 (1917).

24. Rasmussen, H. W. Cretaceous Crinoidea (Comatulida and Roveacrinida) from England and France. *Bulletin of the Geological Society of Denmark* **20**, 285–294 (1971).

25. Tobin, T.S. et al. Extinction patterns, δ18O trends, and magnetostratigraphy from a southern high-latitude Cretaceous–Paleogene section: links with Deccan volcanism. *Palaeogeogr Palaeoclimatol Palaeoecol* **350**, 180–188 (2012).

26. Macellari, C. E. Stratigraphy, sedimentology, and paleoecology of Upper Cretaceous/Paleocene shelf-deltaic sediments of Seymour Island. *Geological Society of America Memoirs* **169**, 25–54 (1988).

27. Marenssi, S., Santillana, S. & Bauer, M. Estratigrafía, petrografía sedimentaria y procedencia de las formaciones Sobral y Cross Valley (Paleoceno), isla Marambio (Seymour), Antártica. *Andean Geology* **39**, 67–91 (2012).

28. Crame, J. A., Francis, J. E., Cantrill, D. J. & Pirrie, D . Maastrichtian stratigraphy of Antarctica. *Cretac Res* **25**, 411–423 (2004).

29. Tambussi, C. P., Reguero, M. A., Marenssi, S. A. & Santillana, S. N. *Crossvallia unienwillia*, a new Spheniscidae (Sphenisciformes, Aves) from the Late Paleocene of Antarctica. *Geobios* **38**, 667–675 (2005).

30. Elliot, D. H. & Trautman, T. A. Lower Tertiary strata on Seymour Island, Antarctic Peninsula. *Antarctic Geoscience*, ed Craddock, C. (University of Wisconsin Press, Madison), pp 287–297 (1982).

31. Askin, R. A. Campanian to Paleocene palynological succession of Seymour and adjacent islands, northeastern Antarctic Peninsula. *Geological Society of America Memoirs* **169**, 131–154 (1988).

32. Wrenn, J. H. & Hart, G. F. Paleogene dinoflagellate cyst biostratigraphy of Seymour Island, Antarctica. *Geology and Paleontology of Seymour Island, Antarctic Peninsula. Geological Society of America Memoirs* **169**, 321–448 (1988).

33. Doktor, M. et al. Argentine-Polish geological investigations on Seymour (Marambio) Island. *Polish Polar Research* **9**, 521–541 (1988).

34. Hocking, R.M., Moors, H. T. & Van de Graaff, W. J. E. Geology of the Carnarvon Basin Western Australia. *Bulletin of Geological Survey Western Australia* **133**, 1–289 (1987).

35. Bromley, R. G. Marine phosphorites as depth indicators. *Mar Geol* **5 (5-6)**, 503–509 (1967).

36. Porrenga, D. H. Glauconite and chamosite as depth indicators in the marine environment. *Mar Geol* **5 (5-6)**, 495–502 (1967).

37. Clarke, J. D. A., Gammon, P. R., Hou, B. & Gallagher, S. J. Middle to Upper Eocene stratigraphic nomenclature and deposition in the Eucla Basin. *Australian Journal of Earth Sciences* **50**, 231–248 (2003).

38. McGowran, B. et al. Australasian palaeobiogeography: the Palaeogene and Neogene record. *Palaeobiogeography of Australasian Faunas and Floras. Association of*

Australasian Palaeontologists Memoir 23, eds Wright, A. J., Young, G. C., Talent, J. A., & Laurie, J. R.), pp 405–470 (2000).

39. Cockbain, A. E. The stratigraphy of the Plantagenet Group, Western Australia. *Geological Survey Western Australia Annual Report 1967*, 61–63 (1968).
40. Singleton, O. P. The Tertiary stratigraphy of Western Australia, a review. *Pan Indian Ocean Science Congress, Perth, 1954, Section C*, pp 59–65 (1954).
41. Li, Q., James, N. P., Bone, Y. & McGowran, B. Foraminiferal biostratigraphy and depositional environments of the mid□Cenozoic Abrakurrie Limestone, Eucla Basin, southern Australia. *Australian Journal of Earth Sciences* **43**, 437–450 (1996).
42. Reynolds, M. A. The Cainozoic succession of Maslin and Aldinga Bays. *Transactions of the Royal Society of South Australia* **76**, 114–140 (1951).
43. Majoran, S. Late Eocene ostracode biofacies of the Tortachilla Limestone, and the Tuketja Member of the Blanche Point Formation, South Australia. *GFF* **117**, 75–80 (1995).
44. Jenkins, R. J. F., Jones, J. B., McGowran, B., Beecroft, A. S. & Fitzgerald, M. J. Lithostratigraphic subdivision of the Blanche Point Formation, Late Eocene, Willunga sub-basin. *Quarterly Geological Notes, Geological Survey of South Australia* **84**, 2–7 (1982).
45. McGowran, B. & Beecroft, A. Foraminiferal biofacies in a silica-rich neritic sediment, Late Eocene, South Australia. *Palaeogeogr Palaeoclimatol Palaeoecol* **52 (3-4)**, 321–346 (1986).
46. Ludbrook, N. H. Correlation of the Tertiary rocks of South Australia. *Transactions of the Royal Society of South Australia* **87**, 5–15 (1963).
47. Majoran, S. Palaeobathymetry of ostracod associations before and after the Chinaman Gully regression ('Eocene/Oligocene boundary') in South Australia. *Alcheringa* **20**, 245–267 (1996).

48. Lindsay, J. M. Aspects of South Australian Tertiary foraminiferal biostratigraphy, with emphasis on studies of Massilina and Subbotina. *South Australia Department of Mines and Energy Special Publication* **5**, 187–231 (1985).

49. Cooper, B. J. The Cainozoic St Vincent Basin–tectonics, structure, stratigraphy. *Stratigraphy, palaeontology, malacology: papers in honour of Dr Nell Ludbrook. Department of Mines and Energy, South Australia, Special Publication* **5**, 35–49 (1985).

50. McKenzie, K. G. & Cooper, B. J. Appendix 2. Notes on Ostracoda from Willunga Embayment boreholes WLG38, WLG40 and WLG42. *Eocene to Miocene stratigraphy of the Willunga Embayment. Geological Survey of South Australia, Report of Investigations*, ed Cooper, B. J. (Geological Survey of South Australia, Adelaide), Vol 50, pp 90–101 (1979).

51. McKenzie, K. G., Reyment, R. A. & Reyment, E. R. Eocene-Oligocene Ostracoda from South Australia and Victoria, Australia. *Revista Española de Paleontologia* **6**, 135–175 (1991).

52. Neil, J. V. Comparisons between some Middle Miocene and Recent southeastern Australian ostracode assemblages. *Ostracoda in the Earth and Life Sciences. Balkema, Rotterdam*, eds McKenzie, K. G. & Jones, P. J. (A. A. Balkema, Rotterdam), pp 277–290 (1993).

53. Lukasik, J. J. & James, N. P. Lithostratigraphic revision and correlation of the Oligo□ Miocene Murray Supergroup, Western Murray Basin, South Australia. *Australian Journal of Earth Sciences* **45**, 889–902 (1998).

54. Lukasik, J. J., James, N. P., McGowran, B. & Bone, Y. An epeiric ramp: low-energy, cool-water carbonate facies in a Tertiary inland sea, Murray Basin, South Australia. *Sedimentology* **47**, 851–881 (2000).

55. Shafik, S. The significance of some subtle changes in the composition of Late Cretaceous and early Tertiary nannofloras from Australia. *INA Newsletter* **15**, 93–94 (1993).

56. Haq, B. U., Hardenbol, J. & Vail, P. R. Mesozoic and Cenozoic chronostratigraphy and cycles of sea-level change. *Sea-Level Changes: An Integrated Approach, Edition: SEPM Special Publication 42*, eds Wilgus, C. K., Hastings, B. S., Kendall, C. G., Ross, C. A. & van Wagoner, J. C. (SEPM, Tulsa, Oklahoma, USA), pp 7–108 (1988).

57. Shafik, S. Calcarcous microplankton biostratigraphy of the Eocene Browns Creek Clay in the Aire District, Otway Basin of southeastern Australia: An update. *AGSO Journal of Australian Geology and Geophysics* **16**, 333–344 (1995).

58. Tickell, S. J., Abele, C. & Edwards J. Port Campbell Embayment: 1: 100 000 map geological report. *Geological Survey of Victoria, Report* 95 (1992).

59. Abele, C. Late Eocene and the Eocene/Oligocene boundary in the Aire District, Victoria. *Geological Survey of Victoria, Unpublished Report* **7**, 1–31 (1994).

60. Shafik, S. & Idnurm, M. Calcareous microplankton and polarity reversal stratigraphies of the Upper Eocene Browns Creek clay in the Otway Basin, Southeast Australia: Matching the evidence. *Australian Journal of Earth Sciences* **44**, 77–86 (1997).

61. Hutton, F. W. *Catalogue of the Tertiary Mollusca and Echinodermata of New Zealand: In the collection of the Colonial Museum* (New Zealand Geological Survey, Wellington) p 48 (1873).

62. Cox, S. & Barrell, D. J. A. *Geology of the Aoraki Area, New Zealand*. Institute of Geological and Nuclear Sciences 1:250,000 geological map 15. Lower Hutt (New Zealand) New Zealand Institute of Geological and Nuclear Sciences Limited (2007).

63. Forsyth, P. J., Barrell, D. J. A. & Jongens R (compilers) *Geology of the Christchurch area*. Institute of Geological and Nuclear Sciences 1:250 000 geological map 16. 1 sheet + 67 p.

Lower Hutt (New Zealand) New Zealand Institute of Geological and Nuclear Sciences Limited (2008).

64. Rattenbury, M. S., Townsend, D.B. & Johnston, M. R. *Geology of the Kaikoura area*. 1:250000 Geological map 13. Lower Hutt (New Zealand) New Zealand Institute of Geological and Nuclear Sciences Limited (2006).
65. Eagle, M. K. New Fossil Crinoids (Articulata: Comatulida) from the Late Oligocene of The Pentland Hills and Hurstlea, South Island. *Records of the Auckland Museum* **44**, 85–110 (2007).
66. Eagle, M. K. A new fossil isocrinid crinoid from the Late Oligocene of Waitete Bay, northern Coromandel. *Records of the Auckland Institute and Museum* **30**, 1–12 (1993).
67. Robinson, J. H. & Lee, D. E. A shallow, warm-water calcitic molluscan fauna from an Early Oligocene seamount, North Otago, New Zealand. *New Zealand Journal of Geology and Geophysics* **54**, 135–147 (2011).
68. Fordyce, R. E. Late Eocene archaeocete whale (Archaeoceti: Dorudontinae) from Waihao, South Canterbury, New Zealand. *New Zealand Journal of Geology and Geophysics* **28**, 351–357 (1985).
69. Kelly, M., Lee, D., Kelly, S. & Buckeridge, J. S. A recent sponge, Pleroma aotea Kelly (" Order" Lithistida: Family Pleromidae), in the late Eocene Ototara Limestone of Otago, New Zealand. *New Zealand Journal of Marine and Freshwater Research* **37**, 129–148 (2003).
70. Forsyth, P. J.. *Geology of the Waitaki Area*. Institute of Geological and Nuclear Sciences 1:250,000 geological map 19, 1 sheet and 64 pp. Lower Hutt (New Zealand) New Zealand Institute of Geological and Nuclear Sciences Limited (2001).
71. Feldmann, R. M. & Maxwell, P. A. Late Eocene decapod Crustacea from North Westland, South Island, New Zealand. *J Paleontol* **64**, 779–797 (1990).

72. Hay, R. F., Mutch, A.R. & Watters, W. A. Geology of the Chatham Islands. *New Zealand Geological Survey Bulletin* **83**, 1–85 (1970).

73. Sorrentino, L., Stilwell, J. D. & Mays, C. A model of tephra dispersal from an early Palaeogene shallow submarine Surtseyan-style eruption (s), the Red Bluff Tuff Formation, Chatham Island, New Zealand. *Sedimentary Geology* **300**, 86–102 (2014).

74. Campbell, H. J. et al. Cretaceous-Cenozoic geology and biostratigraphy of the Chatham Islands, New Zealand. *Monograph of the Institute of Geological and Nuclear Sciences* **2**, 1–269 (1993).

75. Stilwell, J. D., Fordyce, R. E. & Rolfe, P. J. Paleocene isocrinids (Echinodermata: Crinoidea) from the Kauru Formation, South Island, New Zealand. *J Paleontol* **68**, 135–141 (1994).

76. Eagle, M. K. A new genus of fossil crinoid (Cyrtocrinidia: Sclerocrinidae) from Chatham Island, New Zealand. *Records of the Auckland Museum* **42**, 35–47 (2005).

77. Bishop, D. G. & Turnbull, I. M. *Geology of the Dunedin area*. Institute of Geological & Nuclear Sciences 1: 250 000 Geological Map 21. Lower Hutt (New Zealand) New Zealand Institute of Geological and Nuclear Sciences Limited (1996).

78. Malumián, N. & Olivero, E. B. Shallow-water late middle Eocene crinoids from Tierra del Fuego: a new southern record of a retrograde community structure. *Scientia Marina* **69**, 349–353 (2005).

79. Clyde, W. et al. New age constraints for the Salamanca Formation and lower Río Chico Group in the western San Jorge Basin, Patagonia, Argentina: Implications for Cretaceous-Paleogene extinction recovery and land mammal age correlations. *Geol Soc Am Bull* **126 (3-4)**, 289–306 (2014).

80. Zinsmeister, W. J., Feldmann, R. M., Woodburne, M. O. & Elliot, D. H. Latest Cretaceous/earliest Tertiary transition on Seymour Island, Antarctica. *J Paleontol* **63**, 731–738 (1989).
